# Mechanical stress shapes the cancer cell response to neddylation inhibition

**DOI:** 10.1186/s13046-022-02328-y

**Published:** 2022-03-30

**Authors:** Frédérique Mittler, Patricia Obeïd, Vincent Haguet, Cédric Allier, Sophie Gerbaud, Anastasia V. Rulina, Xavier Gidrol, Maxim Y. Balakirev

**Affiliations:** 1 University Grenoble Alpes, CEA, INSERM, IRIG, Biomics, 38054 Grenoble, France; 2 University Grenoble Alpes, CEA, LETI, 38054 Grenoble, France; 3 University of Bergen, Bergen, Norway

**Keywords:** MLN4924, Rho GTPases, Mechanical stress, Tight junctions, Metastasis, Prostate cancer

## Abstract

**Background:**

The inhibition of neddylation by the preclinical drug MLN4924 represents a new strategy to combat cancer. However, despite being effective against hematologic malignancies, its success in solid tumors, where cell–cell and cell-ECM interactions play essential roles, remains elusive.

**Methods:**

Here, we studied the effects of MLN4924 on cell growth, migration and invasion in cultured prostate cancer cells and in disease-relevant prostate tumoroids. Using focused protein profiling, drug and RNAi screening, we analyzed cellular pathways activated by neddylation inhibition.

**Results:**

We show that mechanical stress induced by MLN4924 in prostate cancer cells significantly affects the therapeutic outcome. The latter depends on the cell type and involves distinct Rho isoforms. In LNCaP and VCaP cells, the stimulation of RhoA and RhoB by MLN4924 markedly upregulates the level of tight junction proteins at cell–cell contacts, which augments the mechanical strain induced by Rho signaling. This “tight junction stress response” (TJSR) causes the collapse of cell monolayers and a characteristic rupture of cancer spheroids. Notably, TJSR is a major cause of drug-induced apoptosis in these cells. On the other hand, in PC3 cells that underwent partial epithelial-to-mesenchymal transition (EMT), the stimulation of RhoC induces an adverse effect by promoting amoeboid cell scattering and invasion. We identified complementary targets and drugs that allow for the induction of TJSR without stimulating RhoC.

**Conclusions:**

Our finding that MLN4924 acts as a mechanotherapeutic opens new ways to improve the efficacy of neddylation inhibition as an anticancer approach.

**Graphical Abstract:**

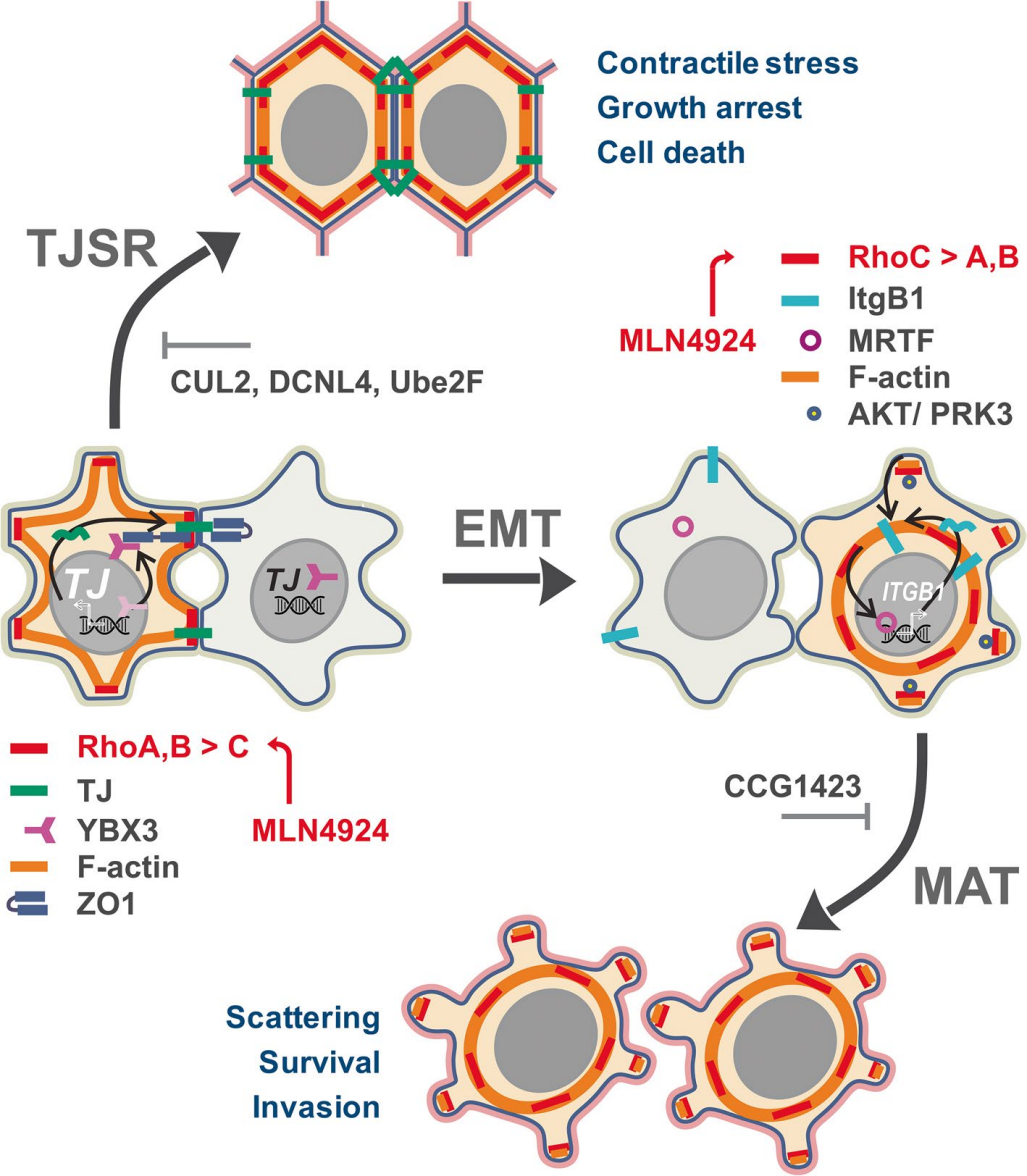

**Supplementary Information:**

The online version contains supplementary material available at 10.1186/s13046-022-02328-y.

## Background

The recurrence of treatment-resistant metastatic diseases is responsible for most cancer-related deaths. This is a result of the relative inefficacy of the current drug discovery strategy that selects compounds mainly for their antiproliferative potencies and abilities to reduce the size of a primary tumor (response evaluation criteria in solid tumors, RECIST) [1]. As a result, chemotherapies fail to prevent metastasis and can even promote cancer cell dissemination by selecting treatment-resistant aggressive phenotypes [2, 3].

In this work, we studied the effects of the preclinical drug MLN4924 (International Nonproprietary Name: Pevonedistat; hereafter called “MLN”) in prostate cancer cells. There are currently 40 (including 14 in solid tumors) phase I-III clinical studies of pevonedistat listed on ClinicalTrials.gov. MLN specifically inhibits Nedd8-activating enzyme (NAE1) and, as a result, blocks neddylation of cellular proteins [4]. This, in turn, prevents the degradation of approximately one-third of the human proteome, which depends on the activity of Nedd8-dependent ubiquitin ligases, of which cullin-RING E3 ligases (CRLs) are the main class [5–7]. The current understanding is that similar to clinically approved proteasome inhibitors, MLN exerts its anticancer activity by stabilizing a number of tumor suppressors and blocking several oncogenic pathways [8].

In various types of cancer cells, including prostate cancer, MLN induces self-inflicted DNA damage via re-replication, cell cycle arrest and apoptosis [4, 9, 10]. A recent study suggested that MLN could also suppress prostate cancer (PCa) cells specifically by shutting down the transcription of the androgen receptor (AR) and its downstream targets [11]. On the other hand, we have shown that in VCaP cells bearing amplified copies of the AR gene, MLN could stimulate AR transcription and promote cell survival, particularly upon androgen depletion [12]. Notably, in VCaP cells, MLN markedly stimulates the prometastatic Wnt/β-Cat–FoxO pathway [12]. In androgen-independent PC3 cells, MLN was shown to promote cell proliferation and tumor sphere formation [13] and to accelerate cancer cell migration [14]. The investigation of these adverse drug effects is essential for further development of neddylation inhibitors as anticancer therapeutics.

Herein, we show that mechanical stress induced by MLN in cancer cells is an important determinant of the therapeutic outcome. Depending on the cell type and the prevalence of Rho GTPase signaling, MLN treatment results in cell clustering or invasive cell scattering. Notably, it also controls cancer cell survival. We analyzed cellular pathways involved in this regulation and identified cancer cell vulnerabilities that help prevent adverse effects and improve the efficacy of neddylation inhibition.

## Methods

Complete list of materials and experimental details as well as all Supplementary Figures and Tables are given within the Additional File 1 (Supplementary Information).

### Cell culture

All cell lines were purchased from the American Type Culture Collection (ATCC), cultured as recommended, and tested on a semester basis. Spheroid culture and analysis were performed as previously described [15]. Tumoroids were grown from a single cell suspension on the solidified Matrigel bed (50 µl, 7 mg/ml Matrigel in PBS) in 96-well plates. The growth medium was supplemented with 0.4 mg/ml Matrigel.

### Soft agar colony formation assay

The assay was performed in 6-well plates according to standard procedures. Cells were incorporated in 0,35% low-melting-point agarose gel and grown for > 2 weeks to generate colonies. For “pulse” regimen, the cells were pre-treated with 100 nM MLN for 1 h in suspension before mixing with the agarose solution. The colonies were stained with Nitro Blue Tetrazolium (NBT, Sigma-Aldrich). The images were acquired with ChemiTouch (Bio-Rad) and analyzed using ImageJ software.

### Wound healing assay

The assay was performed in 96-well plates. Cell monolayers were scratched using a wound replicator equipped with 96 stainless steel pins (V&P Scientific, San Diego, California). The imaging was performed within the cell culture incubator on in-line holographic microscope equipped with 96 quasi-coherent light sources and image sensors. The images were quantified using ImageJ software.

### ATP-based viability assay

Cell metabolism was analyzed by measuring ATP content using ViaLight™ Plus Cell Proliferation and Cytotoxicity BioAssay Kit (Lonza) essentially as described [12, 15].

### Microscopy & flow cytometry

Immunofluorescence microscopy was performed in 96-well black/clear plates using Axioimager Z1 Apotome fluorescence microscope (Zeiss). The antibodies are listed in Supplementary Table S2. Analysis of C-CPE binding to PCa cells was performed both in live and fixed cells using Cy3-labeled C-CPE protein. Flow cytometry was performed on the BD LSR II flow cytometer (BD Biosciences). Cell, spheroid, and tumoroid morphologies as well as cell apoptosis were analyzed by automated microscopy on CellInsight NXT High Content Screening Platform (Thermo Scientific) as described [12, 15].

### Western blotting, small GTPase assay, and immunoprecipitation

Standard procedures were used for western blotting. The analysis of small GTPases was performed by selective capturing GTP-bound forms of GTPases on the beads coated with the affinity ligands: Rhotekin Rho Binding Domain (RBD) and the Cdc42- and Rac-Interactive Binding motif (CRIB). Immunoprecipitations were performed using ZO1 antibody covalently coupled to NHS Mag Sepharose beads (Cytiva) essentially as described [16]. The antibodies are listed in Supplementary Table S2.

### Luciferase Reporter Assay and qPCR

Luciferase Reporter Assay and qPCR were performed essentially as described [12]. CLDN4 Firefly luciferase reporter [17] and a constitutively-active Renilla luciferase reference vector were used.

### Drug and siRNA screens

Both screens were performed in 6-well plates as described [12]. The Clnd4 expression was analyzed by western blotting and quantified using ImageJ software. The lists of the drugs and siRNAs are given in Supplementary Tables S1 and S3, respectively.

## Results

### MLN induces distinct phenotypes in PCa cells

We studied the effects of neddylation inhibition in LNCaP, PC3, and VCaP prostate cancer cells. These cell lines have very different genotypes and phenotypes, representing the heterogeneity of prostate cancer [18]. Previously, we found that MLN inhibits neddylation in these cell lines with EC50s < 50 nM [12]. Therefore, in the present work, the drug was used at concentrations below 500 nM to avoid unspecific effects.

**Fig. 1:**
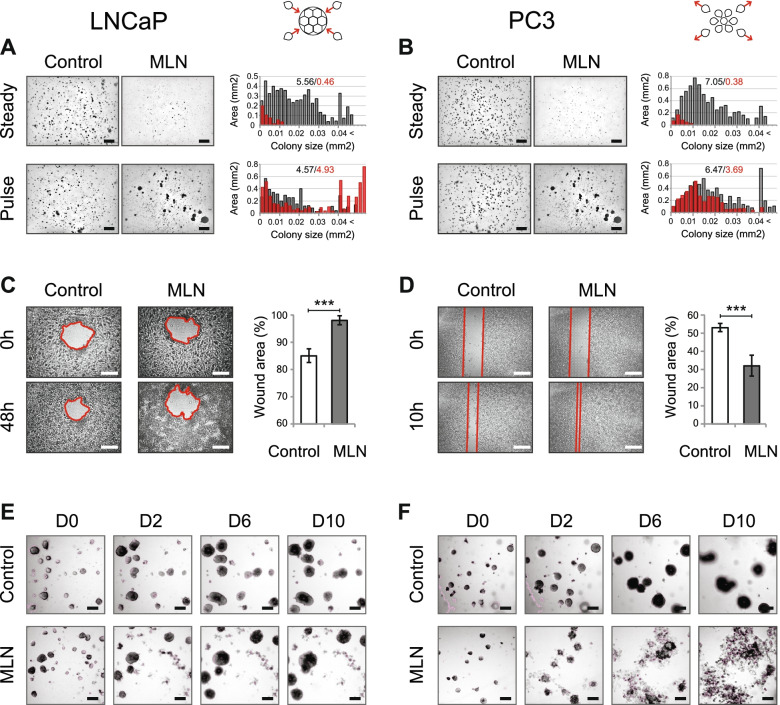
Neddylation inhibition induces distinct phenotypes in PCa cells. **A**, **B** Effect of 100 nM MLN on PCa colony growth in soft agar (“steady”). In the “pulse” regimen, the cells were pretreated with 100 nM MLN for 1 h before seeding (see also Supplementary Figure S1A). The histograms show the colony size distribution of control (black)- and MLN (red)-treated cells. The numbers indicate the total area occupied by the colonies of the given size. Scale bar = 1 mm. **C**, **D** Wound healing assay with LNCaP (**C**) and PC3 (**D**) cells (mean ± S.D., n = 5 for LNCaP, *n* = 6 for PC3, ***-*p* < 0.001). 100 nM MLN was added just before monolayer scratching. Scale bar = 500 µm (**E**, **F**) Effect of 100 nM MLN on LNCaP (**E**) and PC3 (**F**) tumoroid growth over 10 days. Scale bar = 150 µm. In all experiments with MLNs, DMSO was used as a vehicle control

Because we showed that that MLN could stimulate the prometastatic Wnt/β-Cat–FoxO pathway [12], we examined its effect on anchorage-independent growth, migration and invasion. In all three cell lines, MLN suppressed cell growth in agarose, reducing both the number and the mean size of the colonies (Fig. 1A, B, “Steady”; Supplementary Figure S1A). A previous study showed that a short MLN treatment was effective in inducing colorectal cancer cell death [9].

We tested the “pulse” regimen by pretreating the cells for 1 h in suspension before seeding in agarose (Fig. 1A, B, “Pulse”; Supplementary Figure S1A). Notably, the cell lines responded differently: in PC3 cells, MLN significantly reduced the total mass and the mean size of the colonies, whereas in LNCaP and VCaP cells, pretreatment resulted in the formation of large cell clusters without significant changes in the total mass. Colony size analysis indicated that LNCaP and VCaP cells were prone to aggregation and formed colonies with polymodal size distributions, whereas the size distribution of PC3 colonies was nearly unimodal (Fig. 1A, B; Supplementary Figure S1A). Notably, MLN pretreatment promoted aggregation in LNCaP and VCaP cells but prevented aggregation in PC3 cells. Further analysis of monolayer cultures by conventional and lens-free microscopy confirmed MLN-induced cell clustering in LNCaP and VCaP cells (Supplementary Figure S1B, C). MLN also significantly accelerated the assembly of VCaP spheroids in U-bottomed plates (Supplementary Figure S1D). Because the latter depends both on cell–cell interactions and cell migration [15], we examined the effect of the drug on cell migration using a wound healing assay (Fig. 1C, D; Supplementary Figure S2A, B). MLN treatment resulted in the arrest of LNCaP cell migration and retraction of the monolayer into individual clusters. Microscopic examination revealed that MLN blocked the formation of cell protrusions and polarized cell migration into the wound (Supplementary Figure S2A). In contrast, and in agreement with a previous report [14], MLN at doses ≤ 100 nM significantly stimulated the migration of PC3 cells (Fig. 1D; Supplementary Figure S2B). PC3 monolayers treated with MLN displayed loose leading edges with many scattered poorly polarized cells (Supplementary Figure S2B).

Therefore, neddylation inhibition results in two distinct cell responses. In LNCaP and VCaP cells, MLN stimulates cell–cell interactions and clustering while inhibiting cell migration. In PC3 cells, MLN increases cell migration and scattering. Because both responses are implicated in cancer invasion and metastasis [19], we investigated the effect of MLN in disease-relevant Matrigel cultures grown from a suspension of single cells (hereafter called “tumoroids” to distinguish them from “spheroids” grown without extracellular matrix (ECM) in U-bottomed plates; Fig. 1E, F; Supplementary Figure S2C, D). Although MLN suppressed the growth of both LNCaP and PC3 tumoroids, time-lapse microscopy revealed very different responses. In LNCaP cells, MLN induced disintegration of some tumoroids, while leaving the others unaffected (Fig. 1E). The latter continued to grow at a pace similar to that of the control, often swallowing smaller tumoroids and their remnants (Supplementary Figure S2C). The effect of MLN in PC3 cells was strikingly different, resulting in significant tumoroid disassembly (Fig. 1F; Supplementary Figure S2D). Importantly, the scattered cells continued to proliferate and spread, suggesting resistance to drug treatment. Microscopic examination revealed a heterogeneous population of rounded cells and oversized cells with large nuclei, the defining characteristics of polyploid giant cancer cells (Supplementary Figure S2D, S9E) [20]. Thus, the response of PC3 tumoroids to MLN treatment resembles metastatic spread with the appearance of therapeutically resistant cancer cells.

### MLN upregulates RhoA and stabilizes F-actin

The drastic changes in cell morphology and motility induced by MLN indicate the involvement of the actomyosin cytoskeleton. The inhibition of cellular neddylation has been shown to stimulate RhoA signaling and induce mechanical stress [21, 22]. We observed that MLN upregulates RhoA and activates the RhoA/ROCK/Cofilin pathway in all three PCa cell lines (Supplementary Figure S3). Notably, stabilization of actin filaments occurred both in LNCaP/VCaP cell clusters and in scattered PC3 cells, suggesting that the activation of RhoA alone cannot explain the differences in cell responses.

### MLN affects distinct sets of membrane proteins in PCa cells

**Fig. 2:**
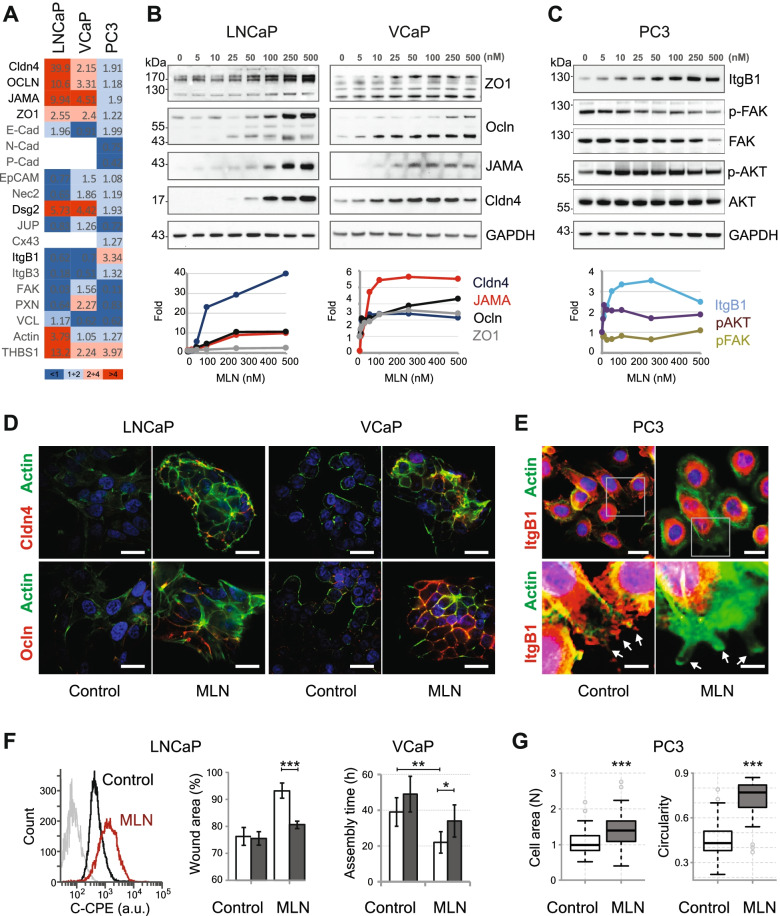
MLN affects distinct sets of membrane proteins in PCa cells. **A** Effect of MLN on protein expression in PCa cells measured by western blotting. The numbers indicate the fold increase compared to the control. **B** Western blots show upregulation of TJ proteins in LNCaP and VCaP cells with the corresponding quantification below. **C** Effect of MLN on ItgB1 and related signaling in PC3 cells. **D** Cell morphology and expression of Cldn4 and Ocln proteins in LNCaP and VCaP cells treated with 100 nM MLN. Scale bar = 25 µm. **E** Expression of ItgB1 in PC3 cells treated with 100 nM MLN. Actin-positive protrusions in MLN-treated cells showed no ItgB1 staining compared to the control (white arrows). Scale bar = 25 µm and 10 µm (zooms). **F** On the left: binding of Cy3-labeled C-CPE to control and 100 nM MLN-treated LNCaP cells analyzed by flow cytometry. The gray trace corresponds to the control cells not exposed to Cy3-C-CPE. In the middle: effect of 200 µg/ml C-CPE on wound closure by control and 100 nM MLN-treated LNCaP cells. The C-CPE conditions are shown in dark gray. On the right: effect of 200 µg/ml C-CPE on spheroid assembly by control and 100 nM MLN-treated VCaP cells. The C-CPE conditions are shown in dark gray. **G** Effect of 100 nM MLN on PC3 cell morphology measured by automated microscopy. In all experiments, the cells were treated with MLN for 20 h with DMSO as a vehicle control. Statistical significance: *- *p* < 0.05, **-*p* < 0.01 and ***-*p* < 0.001

In stem cells, RhoA activity [23] or suppression of the Nedd8-dependent cullin 3 ligase CRL3 [24] induces cell clustering. This phenotype depends on the abundance of proteins such as E-cadherin (E-Cad) and collagen [23, 24]. Using western blotting, we examined the effect of MLN on a panel of proteins involved in cell–cell and cell-ECM interactions in PCa cells (Fig. 2A; Supplementary Figure S4). Only mild differences were found in E-Cad levels that did not correlate with cell phenotypes. Nevertheless, the overall response of LNCaP cells clearly differed from that of PC3 cells, and VCaP showed an intermediate phenotype (Fig. 2A).

The major differentially regulated proteins were tight junction (TJ) components claudin 4 (Cldn4), occludin (Ocln), JAMA and ZO1 and the desmosomal protein desmoglein 2 (Dsg2), which were markedly upregulated in LNCaP and VCaP cells compared to PC3 cells (Fig. 2A, B; Supplementary Figure S4). On the other hand, PC3 cells showed a significant increase in integrin β1 (ItgB1) expression, whereas a decrease was observed in LNCaP and VCaP cells (Fig. 2A, C; Supplementary Figure S4). Subsequent analysis revealed that the increase in ItgB1 in PC3 cells was mostly due to accumulation of the nonglycosylated form of the receptor, while the level of the mature form was slightly decreased (Supplementary Figure S5A). Consistently, the activity of ItgB1-associated focal adhesion kinase (FAK), as detected by its autophosphorylation at Y397, was only slightly affected while the total level of the protein was decreased (Fig. 2C; Supplementary Figure S5A).

Immunofluorescence analysis of LNCaP and VCaP cell clusters revealed a constricted epithelial architecture with TJ proteins accumulating at the membrane and colocalizing with actin (Fig. 2D; Supplementary Figure S5C). Thus, the mechanical stress induced by MLN seems to partially reverse the cancer phenotype. Notably, the accumulation of TJ proteins at cell–cell contacts appears to contribute to the morphological changes in LNCaP and VCaP cells caused by neddylation inhibition. Indeed, the inhibition of Cldn4-dependent cell–cell adhesion by using its specific ligand *Clostridium perfringens* enterotoxin (C-CPE) [25] overcame the motility arrest induced by MLN in LNCaP cells and significantly delayed the assembly of VCaP spheroids (Fig. 2F; Supplementary Figure S2A, S5D, E).

In contrast, in PC3 cells, MLN induced a poorly polarized phenotype with multiple short membrane protrusions (Fig. 2E; Supplementary Figure S5B). Quantification of the cell morphology revealed an increased cell size and circularity (Fig. 2G). Consistent with the western blot results, MLN treatment resulted in relocalization of ItgB1 from the membrane leading edges to the cytoplasmic compartments and cell cortex (Fig. 2E; Supplementary Figure S5B). Despite this fact and the decrease in FAK activity, MLN-treated PC3 cells showed a significant stimulation of AKT(S473, T308) kinase and prometastatic PRK3/PKN3(T718) [26, 27] (Fig. 2C; Supplementary Figure S5A). These results suggest that MLN induces mesenchymal-to-amoeboid transition (MAT) [28–30] in PC3 cells, which underlies the increase in cell migration and dissemination (Fig. 1D, F, Supplementary Figure S2B).

### Rho activation by MLN triggers the TJ stress response via a YBX3-dependent pathway

**Fig. 3:**
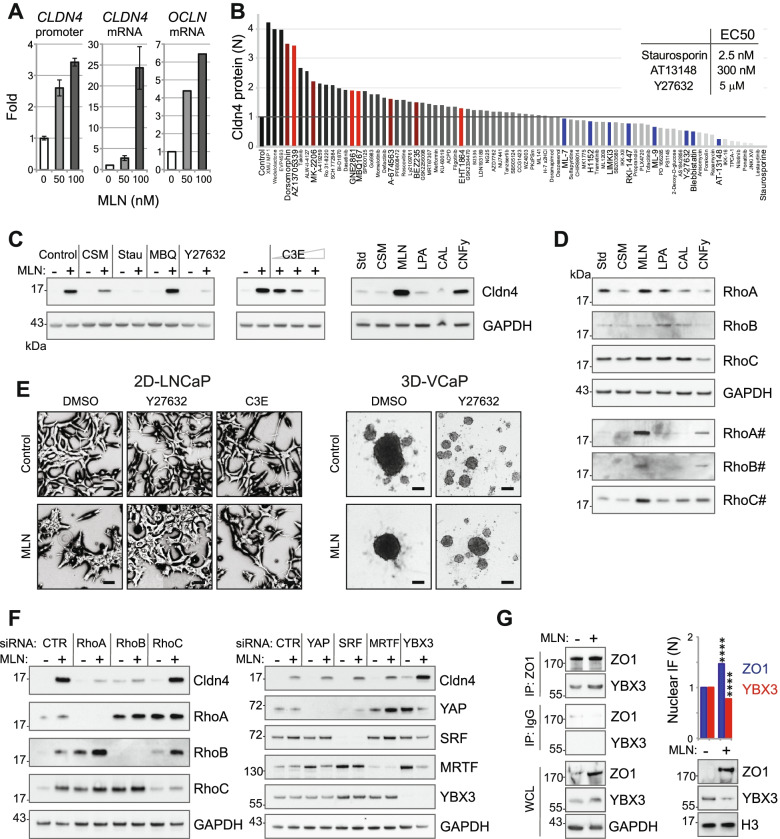
Rho activation by MLN stimulates TJ expression. **A** Effect of MLN on TJ transcription measured by *CLDN4* promoter luciferase reporter and RT–qPCR of *CLDN4* and *OCLN* transcripts in LNCaP cells. **B** Drug effect on the stimulation of Cldn4 expression by MLN (100 nM in control). Inhibitors of PI3K/AKT kinase are indicated in dark red, those of Roc/Cdc42 are indicated in red, and those of Rho signaling are indicated in blue. **C** Rho signaling is required for the stimulation of Cldn4 expression by MLN. Conditions: 100 nM MLN, charcoal-stripped serum (CSM), 20 nM staurosporine (Stau), 5 µM MBQ167 (MBQ), 10 µM Y27632, 5–10–20 µg/ml C3E, 10 µM lysophosphatidic acid (LPA), 50 µM calpeptin (CAL), and 1 nM CNFy. **D** Effect of the drugs (see above) on individual Rho isoforms. The active GTP-bound isoforms are hash-tagged. **E** The effect of 100 nM MLN on LNCaP cell morphology and VCaP spheroid assembly was reversed by 10 µM Y27632 and 20 µg/ml C3E. Scale bars = 50 µm (LNCaP) and 200 µm (VCaP). **F** Effect of knocking down Rho isoforms and specific mechanoresponsive transcriptional regulators on the stimulation of Cldn4 expression by 100 nM MLN. **G** Interaction of ZO1 and YBX3 proteins revealed by western blotting after immunoprecipitation. On the right: Effect of 100 nM MLN on the levels of nuclear ZO1 and YBX3 analyzed by immunofluorescence (histogram) and cell fractionation (western blot). Statistical significance: ***-*p* < 0.001

Because TJs play a discriminatory role in the observed phenotypes, we investigated the mechanisms behind TJ upregulation. MLN did not affect Cldn4 and Ocln degradation, which occurs mostly in lysosomes (Supplementary Figure S6A). In contrast, MLN significantly stimulated the transcription of both the *CLDN4* and *OCLN* genes, as revealed by RT–qPCR and luciferase *CLDN4* reporter assays [17] (Fig. 3A). Several factors have been implicated in the regulation of TJ genes, including epigenetic regulation of the transcription factor Sp1, the Wnt/β-Cat pathway and the AR receptor [31]. However, neither chromatin remodeling drugs nor Wnt/β-Cat inhibitors affected the stimulation of Cldn4 expression by MLN (Supplementary Figure S6B). Modulating AR function had minor effects, which are consistent with a reported inhibition of Cldn4 transcription by AR (Supplementary Figure S6C) [32].

Because we found that TJ stimulation by MLN was diminished in charcoal-stripped medium and completely suppressed by staurosporin (Fig. 3C), it seemed that it was dependent on extracellular cues and kinase signaling. We screened a small library of 79 drugs that target the majority of signaling pathways potentially involved in TJ regulation (Fig. 3B; Supplementary Figure S7; Supplementary Table S1). We observed that four structurally different PI3K/AKT inhibitors potentiated the effect of MLN by further increasing the level of Cldn4 protein (Fig. 3B; Supplementary Figure S7).

This suggested that the stimulation of Cldn4 transcription by MLN results from proapoptotic signaling that is antagonized by PI3K/AKT kinases. Specifically, this TJ stress response (TJSR) is not a consequence of the apoptotic program, as it was not affected by p53/caspase modulators (Supplementary Figure S6D). Several drugs blocked TJSR in a dose-dependent manner, and among them were a few inhibitors of immune NF-κB and JAK signaling pathways (Fig. 3B; Supplementary Figure S7; Supplementary Table S1). However, because of the promiscuity of protein kinase inhibitors and failure of other drugs targeting the same pathways, the role of immune signaling in TJ stimulation by MLN remains uncertain.

The most consistent results were obtained by altering small GTPase signaling with inhibitors of Rac/Cdc42 stimulation and inhibitors of Rho, which suppressed the MLN effect (Fig. 3B, C). Thus, two ROCK inhibitors, Y27632 and AT13148, effectively suppressed MLN-induced Cldn4 expression, LNCaP cell clustering and the assembly of VCaP spheroids (Fig. 3B, C, E; Supplementary Figure S7B; Supplementary Table S1). Further confirming the involvement of Rho, its specific inhibitor, *Clostridium botulinum* C3 exoenzyme (C3E), suppressed both TJ upregulation and cell clustering induced by MLN (Fig. 3C, E). To examine whether Rho activity alone was sufficient to account for the morphogenic effects of MLN, we used different molecules to stimulate Rho in LNCaP cells. Lysophosphatidic acid and calpeptin, two compounds that transiently stimulate Rho activity, did not significantly increase Cldn4 expression. However, *Yersinia pseudotuberculosis* cytotoxic necrotizing factor (CNFy), a specific long-term activator of Rho signaling, strongly induced Cldn4 protein expression (Fig. 3C, D). Similar to MLN, CNFy also induced cell clustering (Supplementary Figure S8A), suggesting that persistent Rho activation is required to change cell morphology. Consistently, the examination of the Rho-GTP pool after 1 day of treatment revealed that only MLNs and CNFys maintained a high level of active Rho (Fig. 3D). Notably, similar to CNFy [33], the neddylation inhibition activated all three (A, B, C) Rho isoforms and induced RhoA and RhoC translocation to the membrane (Supplementary Figures S8B, C & S12A). MLN also significantly upregulated the levels of total RhoA and RhoC proteins, though the fold increase in active Rho-GTP significantly exceeded the corresponding change at the protein level (Fig. 3D; Supplementary Figure S9). This suggests that MLN might specifically affect ubiqutylation and degradation of active Rho. We used a series of affinity-based techniques to address the effects of MLN and other proteolysis inhibitors on Rho ubiquitylation and stability (Supplementary Figure S9). We did not detect ubiquitylated Rho-GTP species probably because of the limited sensitivity of the method. For total protein, only ubiquitylation of RhoC was detected at the endogenous level (Supplementary Figure S9B). However, when over-expressed, RhoA and, particularly, RhoC were found ubiquitylated. Both proteasome and lysosome inhibitors increased the levels of the ubiquitylated species suggesting that both pathways are involved in Rho degradation. Unexpectedly, MLN not only increased the levels of Rho proteins but also markedly upregulated their ubiquitylation (Supplementary Figure S9C & D). This result suggests that the regulation of Rho by neddylation is more complex than previously anticipated.

Depletion of the individual Rho isoforms by siRNAs revealed that RhoA and, to a lesser extent, RhoB were responsible for MLN-induced Cldn4 stimulation (Fig. 3F). We conclude that the inhibition of neddylation results in long-term activation of RhoA and RhoB, which induces F-actin and stimulates the expression of TJ proteins via a mechanosensitive pathway. Consistently, we found that TJSR coincides with F-actin stabilization, significantly precedes the onset of apoptosis and is insensitive to apoptosis modulators (Supplementary Figure S6D-G).

To obtain more information on the TJSR pathway, we knocked down four major mechanosensitive transcription regulators implicated in Rho signaling: YAP, SRF, MRTF, and YBX3 (Fig. 3F). Only YBX3 depletion had an effect on Cldn4 by significantly upregulating both basal and MLN-induced protein levels. YBX3 depletion also markedly stimulated the transcription of CLDN4 gene, as revealed by RT–qPCR (Supplementary Figure S10D). These results suggest that YBX3 represses TJ expression. YBX3/ZONAB has been discovered as a partner protein of ZO1 that inhibits YBX3 by sequestering it at the membrane and preventing its entry into the nucleus [16]. The inhibitory function of ZO1 depends on actomyosin-generated tensile force that activates the protein through stretching [34, 35]. As we found that MLN increased the level of ZO1 and induced its colocalization with sub-membranous actin (Fig. 2B, Supplementary Figure S4, S8C), we examined its interaction with YBX3. ZO1 protein immunoprecipitation confirmed its binding to YBX3 (Fig. 3G). We also observed partial colocalization of these proteins in MLN-treated LNCaP cells (Supplementary Figure S10C). Notably, immunofluorescence microscopy and cell fractionation revealed that MLN significantly decreased the level of nuclear YBX3 while markedly upregulating nuclear ZO1 (Fig. 3G, Supplementary Figure S10A, B). These findings suggest that mechanical stress triggers TJSR via a positive feedback mechanism, which involves the stimulation of TJ gene expression by ZO1, either by sequestering YBX3 or directly.

To ascertain that the stimulation of TJSR by MLN was caused by neddylation inhibition, we examined the effects of NEDD8 and UBA3 depletion (Supplementary Figure S10D-F). As with MLN, the inhibition of neddylation by RNAi stimulated Cldn4 expression at transcript and protein levels (Supplementary Figure S10D-F). The effects of knockdown were, however, less strong than those of MLN. This could be explained by incomplete suppression of the targeted transcripts and high efficiency of neddylation machinery (Supplementary Figure S10E, F). Corroborating this conclusion, we found that the stimulation of TJSR by NEDD8 and UBA3 depletion was greatly potentiated by a suboptimal dose of MLN (20 nM), which by itself had little effect (Supplementary Figure S10D, E).

### Rho activation by MLN triggers MAT and tumoroid spreading in PC3 cells

**Fig. 4:**
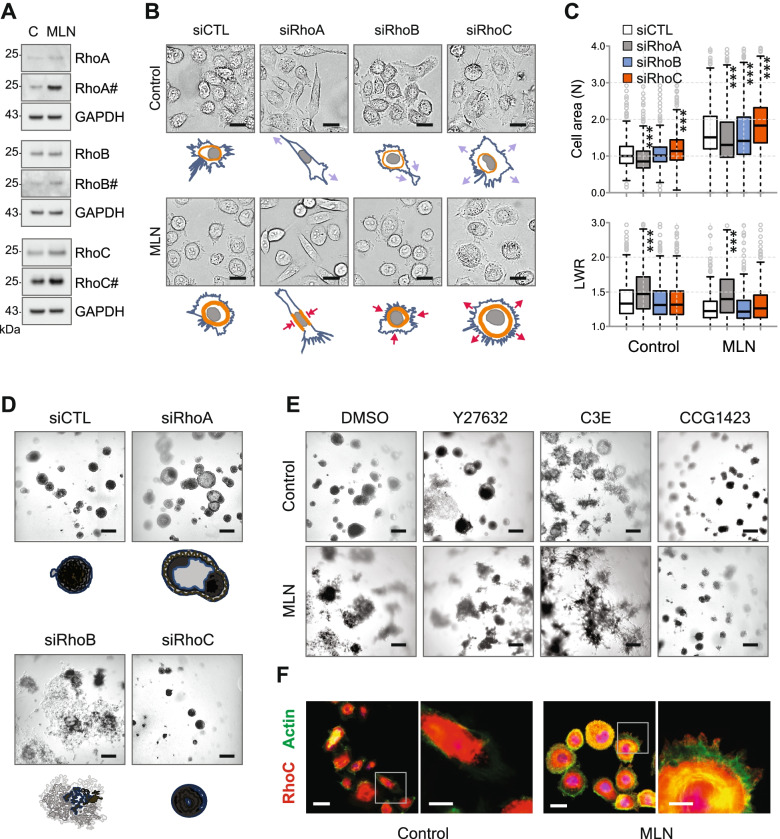
Rho activation by MLN triggers MAT and tumoroid spreading in PC3 cells. **A** Activation of Rho isoforms by 100 nM MLN in PC3 cells. The active GTP-bound isoforms are hash-tagged. **B** Depletion of Rho isoforms in PC3 cells changes cell morphology and affects the cell response to 100 nM MLN. Scale bar = 25 µm. **C** Quantitative analysis of the effect of Rho isoform knockdown shown in (**B**). The cell area and length-to-width ratio (LWR) were measured by automated microscopy. Statistical significance: ***-*p* < 0.001. **D** Effect of the depletion of Rho isoforms on PC3 tumoroid growth. Scale bar = 200 µm. **E** Effect of 10 µM Y27632, 20 µg/ml C3E, and 3 µM CCG1423 on PC3 tumoroid growth with or without (control) 100 nM MLN. Scale bar = 200 µm. **F** Localization of RhoC in control and 100 nM MLN-treated PC3 cells. Scale bar = 25 µm and 10 µm (zooms). In all experiments with MLNs, DMSO was used as a vehicle control

Since Rho signaling and mechanical stress play a central role in the amoeboid phenotype [29, 30], we examined whether it mediates the MAT induced by MLN in PC3 cells. MLN activated all three Rho isoforms in PC3 cells (Fig. 4A). We found that although ItgB1 expression is dependent on MRTF, as previously reported [36], no single Rho isoform is required for its stimulation by MLN (Supplementary Figure S11A, B). Rac1 and Cdc42 have been implicated in SRF/MRTF signaling and, specifically, in the regulation of ItgB1 [37, 38]. However, despite some increase in the basal level of Cdc42 seen in MLN-treated PC3 cells, we did not detect a significant increase in GTP-bound forms of either Rac1 or Cdc42 (Supplementary Figure S11C).

Knocking down individual Rho isoforms revealed opposite effects on cell morphology. RhoA knockdown significantly reduced the cell size and broke the radial symmetry by inducing cell stretching in two opposing directions, whereas RhoC depletion increased the cell size because of radial cell spreading (Fig. 4B, C). Similar results were previously reported and explained by distinct regulation of Rac1 activity by these Rho isoforms [39]. MLN treatment promoted actin cortex thickening and peripheral bundling, which further accentuated the antagonistic effects of isoform knockdown on cell shape, with RhoA/RhoB depletion significantly decreasing, while RhoC depletion increasing cell size (Fig. 4B, C). Therefore, the amoeboid morphology induced by MLN in PC3 cells results from the equilibrium between RhoA/RhoB-dependent radial stretching and RhoC-dependent contractility that prevents excessive expansion of the actomyosin cortex.

Distinct roles of Rho isoforms were confirmed in PC3 tumoroids, where RhoA depletion promoted acinus formation and accelerated the onset of spontaneous invasion, while RhoB depletion greatly stimulated cell dissemination (Fig. 4D). In contrast, the depletion of RhoC suppressed tumoroid growth and completely prevented cell invasion (Fig. 4D). The inhibition of Rho signaling with Y27632 or C3E mimicked RhoA/RhoB depletion, resulting in efficient acinus formation followed by cell escape (Fig. 4E, Supplementary Figure S11D). Invasion was observed in both PC3 and LNCaP tumoroids, which is consistent with a previous report that RhoA/ROCK inhibition stimulates a metastatic switch in PCa cells via a Rac1-dependent mechanism [40]. The latter appears different from the amoeboid cell spreading induced by MLN. Indeed, MLN suppressed both PC3 acinus formation and LNCaP invasion induced by Y27632 and transformed the morphology of spreading PC3 cells from mesenchymal to amoeboid (Fig. 4E; Supplementary Figure S11D, E). On the other hand, and consistent with a previous report [41], suppressing Rho-dependent transcription by CCG1423 mimicked RhoC depletion and efficiently blocked MLN-induced cell scattering (Fig. 4E). Because RhoC is required for amoeboid migration [42, 43], we analyzed the effect of MLN on its localization by immunofluorescence (Fig. 4F). In control cells, the majority of RhoC was found in the cytoplasmic compartments, whereas MLN treatment resulted in significant accumulation of RhoC in the cortex and the plasma membrane. Notably, RhoC colocalized with actin at the distant edges of the blebs, confirming its role in the formation of invasive protrusions.

Knocking down NEDD8 and/or UBA3 reproduced the effects of MLN on PC3 cell morphology with significant stabilization of F-actin, accumulation of RhoC and its translocation to the membrane (Supplementary Figure S12). As with TJSR, these effects were greatly potentiated by a suboptimal dose of MLN (Supplementary Figure S12).

### Rho signaling defines the therapeutic outcome of neddylation inhibition

**Fig. 5:**
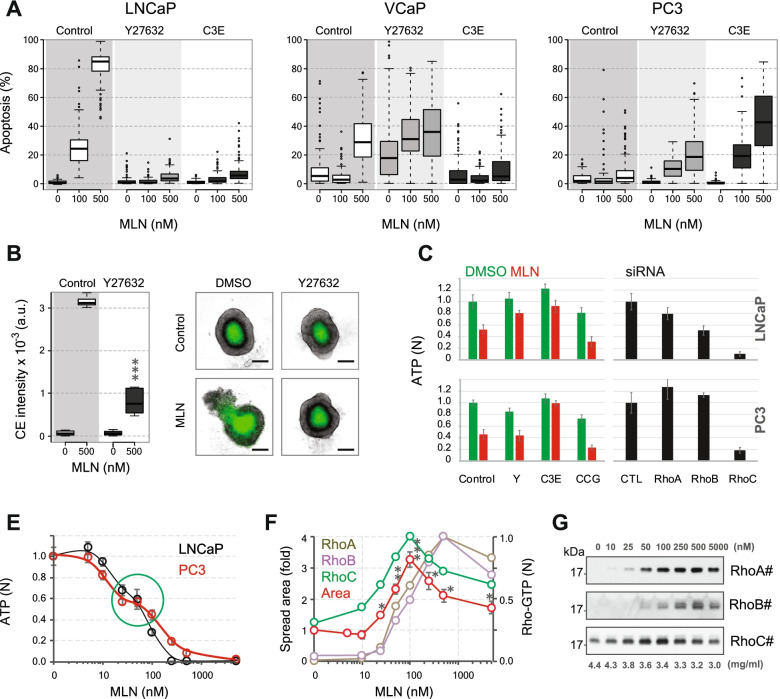
Rho signaling defines the therapeutic outcome. **A** Effect of Rho signaling inhibitors on MLN-induced apoptosis in monolayer cultures of PCa cells. The cells were treated for 1 day with increasing doses of MLN and 10 µM Y27632 or 20 µg/ml C3E. The percentage of apoptotic cells was evaluated by automated fluorescence microscopy using CellEvent™ Caspase-3/7 Green Detection Reagent (CE). For all shown drug vs. control effects: *p* < 0.001 (**B**) Effect of 10 µM Y27632 on MLN-induced apoptosis in LNCaP spheroids. The histogram shows the stimulation of apoptosis by MLN in small (< 250 µm) spheroids measured by CE fluorescence. Statistical significance: ***-*p* < 0.001. On the right: 10 µM Y27632 prevents the mechanical rupture of large (> 400 µm) spheroids induced by 250 nM MLN (the hypoxic apoptotic core of spheroids is stained with CE). Scale bar = 200 µm. **C** Effect of the inhibition of Rho signaling on LNCaP (top) and PC3 (bottom) tumoroid growth. Color histograms: Preformed tumoroids were treated for 7 days with 10 µM Y27632 (Y), 20 µg/ml C3E, or 3 µM CCG1423 with or without (control) 100 nM MLN. The viability was assessed by measuring ATP with ViaLight™ reagent. Black histograms: Tumoroids were grown from Rho-depleted cells for 10 days and assessed for viability. **E** Biphasic MLN dose response curves for LNCaP and PC3 tumoroids. The biphasicity is indicated by the green circle. **F** MLN dose response curves for PC3 tumoroid invasion (measured by tumoroid spread area) and Rho activation. Statistical significance: *- *p* < 0.05, **-*p* < 0.01 and ***-*p* < 0.001. **G** Dose-dependent Rho stimulation by MLN analyzed by western blot and quantified in Fig. 5F. The active GTP-bound isoforms are hash-tagged

Previous studies have shown that RhoA activity could contribute to the therapeutic effects of MLN by blocking tumor-associated angiogenesis [22]. However, it was not considered a primary determinant of the therapeutic outcome. By examining the effect of Rho inhibition on cancer cell survival, we found that both Y27632 and C3E significantly modified the response of cancer cells to MLN (Fig. 5A). The most striking effect was observed in LNCaP cells, where the inhibition of Rho signaling almost completely prevented MLN-induced apoptosis. The ROCK inhibitors Y27632 and AT13148 also blocked apoptosis in small (< 250 µm) LNCaP spheroids and prevented MLN-specific mechanical rupture of large (> 400 µm) LNCaP spheroids [15] (Fig. 5B, Supplementary Figure S13A). Thus, the mechanical stress induced by actomyosin contraction is among the primary causes of MLN-induced death in LNCaP cells. This conclusion is consistent with a previous report that showed high susceptibility of these cells to Rho stimulation by CNFy [33]. In contrast, Rho inhibition in PC3 cells significantly sensitized the cells to MLN-induced apoptosis (Fig. 5A), suggesting a prosurvival function of Rho signaling in PC3 cells. VCaP cells showed an intermediate phenotype with apoptosis stimulated by ROCK inhibition but prevented by suppressing Rho signaling with C3E.

Similar to monolayer and spheroid cultures, Y27632 and C3E reduced MLN toxicity in LNCaP tumoroids (Fig. 5C). Notably, C3E also had an MLN-protective effect in PC3 tumoroids. Thus, contrary to what we observed in monolayer cultures, C3E could have differential specificity toward Rho isoforms in Matrigel. Indeed, acinus formation and cell invasion observed in C3E-treated PC3 tumoroids (Fig. 4E) indicated a preferential inhibition of RhoA/RhoB that did not affect PC3 growth (Fig. 5C). In contrast, suppressing Rho-dependent transcription by CCG1423 blocked invasion and potentiated MLN toxicity in both LNCaP and PC3 tumoroids, which was most consistent with an antiproliferative effect of RhoC inhibition (Fig. 4D and 5C).

These results suggest the opposite roles of Rho isoforms in the therapeutic response of PCa cells to MLN, with RhoA/RhoB being essential for drug toxicity and RhoC protecting against drug toxicity. Notably, this could explain the biphasicity of the MLN dose response observed in 3D cultures of PCa cells that we previously defined as cytostatic versus cytotoxic effects of neddylation inhibition [15]. Thus, in PC3 tumoroids, increasing the MLN dose from 25 to 100 nM did not result in a significant decline in tumoroid viability, while it exerted the highest stimulatory effect on cell invasion (Fig. 5E, F, Supplementary Figure S13B). The latter, in turn, coincided with the maximal level of RhoC stimulation, whereas the activation optima for RhoA/RhoB were shifted to higher MLN doses (Fig. 5F, G). Therefore, biphasicity may result from the peak in RhoC stimulation that promotes cancer cell survival and spread.

### Neddylation-dependent TJ regulators are potential therapeutic targets

**Fig. 6:**
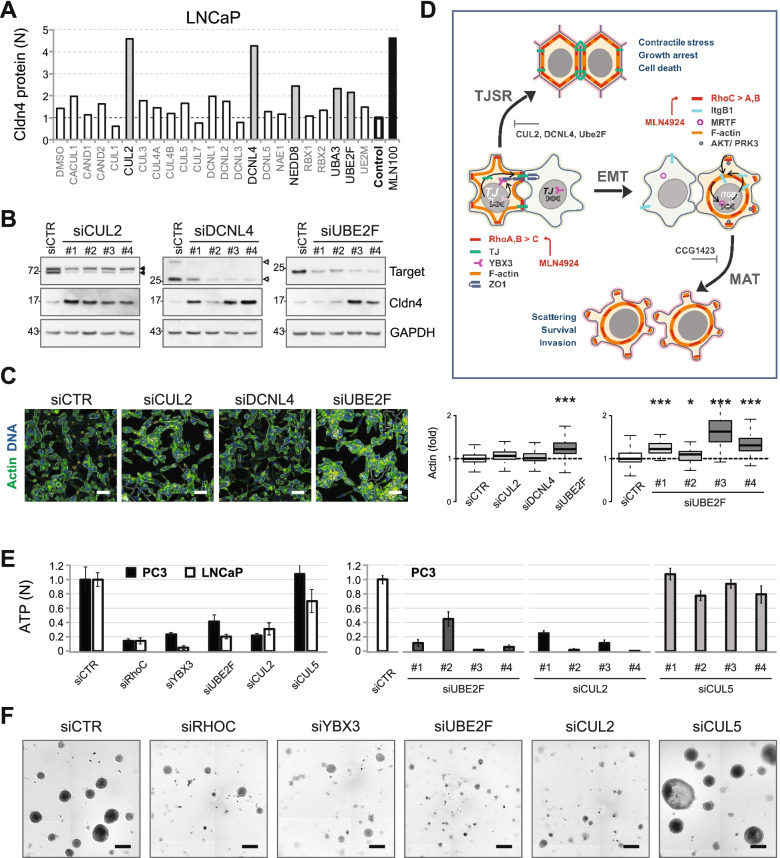
TJSR regulators are potential therapeutic targets. **A** Functional genomic siRNA screen for TJSR regulators in LNCaP cells. The indicated genes were knocked down, and the level of Cldn4 protein was measured by western blot. One hundred nM MLN was used as a reference (MLN100). **B** Hit validation using four individual siRNAs. Western blots show the depletion of the target proteins and the corresponding Cldn4 level. Double black arrowheads indicate native and neddylated CUL2 protein. Double white arrowheads indicate DCNL4 isoforms. **C** Effect of hit depletion on actin polymerization in LNCaP cells. Scale bar = 50 µm. The genes were knocked down using SmartPool siRNAs, and the effect on cell morphology was evaluated by automated fluorescence microscopy and quantified (the histogram in the middle). The histogram on the right shows the validation of the UBE2F hit using four individual siRNAs. Statistical significance: *- *p* < 0.05 and ***-*p* < 0.001. **D** General scheme showing distinct outcomes of neddylation inhibition in PCa cells. **E** Effect of gene knockdown on PC3 and LNCaP tumoroid growth measured with ViaLight™ reagent. The histogram on the right shows the validation of UBE2F and CUL2 hits using four individual siRNAs and comparison with CUL5 in PC3 tumoroids. **F** Effect of gene knockdown on PC3 tumoroid morphology. Scale bar = 200 µm

The results above suggest that in PCa cells, MLN activates two antagonistic Rho-dependent pathways: the activation of RhoA/RhoB stimulates TJSR, inhibits cell migration and induces apoptosis, whereas RhoC mediates prometastatic signaling accompanied by PI3K/AKT/PRK3 activation and promotes MAT, invasion and survival (Fig. 6D). Because these pathways involve distinct regulatory components downstream of NAE1, we aimed to identify neddylation factors specifically involved in TJSR regulation. We performed a functional siRNA screen by knocking down all major genes involved in neddylation. Cldn4 expression was measured as a sensitive and robust indicator of TJSR. In addition to the primary MLN targets UBA3 and NEDD8, the screen identified CUL2, DCNL4, and UBE2F genes, whose suppression increased by more than twice the level of Cldn4 in LNCaP and VCaP cells (Fig. 6A, B and Supplementary Figure S14).

Notably, depletion of neither CUL1 nor CUL3, two bona fide RhoA regulators [44, 45], induced TJSR. Among the hits, only UBE2F knockdown affected cell morphology and stabilized F-actin, whereas CUL2 and DCNL4 probably acted downstream of actin stabilization (Fig. 6C). Depletion of UBE2F and CUL2 suppressed PCa cell proliferation and tumoroid growth with an efficacy comparable to that of RhoC knockdown (Fig. 6F). Although the function of UBE2F is considered CRL5-specific, CUL5 knockdown neither induced a significant increase in Cldn4 expression (Fig. 6A, Supplementary Figure S14A) nor affected tumoroid growth (Fig. 6E). Instead, in PC3 cells, the depletion of CUL5 led to the formation of large acini similar to that seen with RhoA knockdown (Fig. 4D and 6F). These results indicate that UBE2F may function independently of CRL5. Finally, depleting another TJ regulator, YBX3, also blocked the growth and invasion of prostate tumoroids (Fig. 6E, F). Therefore, neddylation-dependent TJ regulators could represent promising therapeutic targets.

## Discussion

Ongoing clinical studies demonstrate the efficacy of neddylation inhibition against hematologic malignancies, which are mostly proliferative diseases. However, success in solid tumors, where cell–cell and cell-ECM interactions play essential roles, remains elusive. By studying neddylation inhibition in PCa cells, we found that cell–cell contacts and Rho signaling significantly affect the therapeutic outcome. Thus, in LNCaP and VCaP cells, MLN effectively induces TJSR and suppresses cell growth, whereas in PC3 cells characterized by partial EMT, it promotes scattering and invasion (Fig. 6D). Notably, these responses involve distinct Rho isoforms.

We show that MLN stimulates all Rho isoforms by a yet to be ascertained mechanism. The stimulation of RhoA by MLN may be a result of the inhibition of one of the known RhoA-specific ubiquitin ligases, (CRL3-BACURD, CRL1-FBXL19/FBXW7 and Smurf-1) [44–46] (see also Supplementary Table S4). Similar mechanisms for RhoB regulation involve CRL2, CRL3-KCTD10 and Smurf-1 ligases [44, 45, 47]. Notably, the fold increase in active Rho-GTP that we observed significantly exceeded the corresponding change at the protein level (Fig. 3D; Supplementary Figure S9). This suggests that Rho stimulation by MLN is a post-translational event and that Rho activation and degradation are coupled. Thus, MLN could stabilize Rho-GTP by preventing its ubiquitylation and subsequent degradation. Curiously, despite the fact that all reported Rho-specific ubiquitin ligases are Nedd8-dependent (Supplementary Table S4), the effect of MLN on Rho ubiquitylation has been studied only for RhoB [47]. Moreover, only a few studies have unambiguously demonstrated Rho ubiquitylation in cells by isolating the proteins in denaturing conditions (Supplementary Table S4). By using this technique, we unexpectedly found that instead of inhibition, MLN treatment markedly upregulated the levels of ubiquitylated RhoA and RhoC (Supplementary Figure S9C, D). Therefore, the regulation of Rho stability and function is complex and implicates both NEDD8-dependent and -independent ubiquitin ligases. Finally, yet another level of Rho regulation by neddylation may involve Rho guanine nucleotide exchange factors [48].

Neddylation inhibition markedly upregulates the expression of TJ proteins in LNCaP/VCaP cells that bolster cell–cell contacts and accentuate the mechanical stress produced by Rho signaling. This causes the collapse of cell monolayers and characteristic rupture of spheroids, leading to apoptosis. These effects are antagonized by ROCK inhibitors and, therefore, are dependent on actomyosin contractility. Interestingly, a recent study demonstrated that actin dynamics could also be regulated by site-specific neddylation of cofilin [49]. Thus, actomyosin cytoskeleton emerges as one of the main effectors of the neddylation pathway.

Because TJSR depends on RhoA/RhoB and implicates YBX3 and ZO1, we propose a model (Fig. 6D) in which prejunctional tension generated by Rho signaling causes a conformational change in ZO1 [34, 35] that allows its binding to YBX3, thus derepressing TJ transcription. The model suggests that YBX3 acts as a transcriptional repressor for TJ genes, which is consistent with our results (Fig. 3F) and previously published data [50, 51]. YBX3/ZONAB can both activate and repress gene expression, the former function being mostly involved in the stimulation of cell proliferation and survival [52], whereas the latter regulates the permeability of the TJ barrier [16]. YBX3/ZONAB binding to ZO1 and sequestration at TJs prevents its transcriptional activity in a cell density-dependent manner [16]. We confirmed that YBX3 and ZO1 interact in LNCaP cells (Fig. 3G). Notably, the inhibition of neddylation increased the levels of both membrane and nuclear ZO1, while the level of nuclear YBX3 significantly decreased (Fig. 3G, Supplementary Figure S8C, S10A, B). These results suggest that the stimulation of TJSR by prejunctional Rho activity involves the suppression of YBX3 function by ZO1.

Compared to LNCaP and VCaP cells, PC3 cells demonstrate a partial EMT phenotype with characteristic expression of vimentin and N-Cad markers (Fig. 2A; Supplementary Figure S4) [53]. PC3 cells possess compromised cell–cell contacts manifested by their inability to form spheroids [15]. This may explain why PC3 cells are unable to initiate TJSR but instead undergo MAT upon neddylation inhibition. Although all Rho isoforms have been implicated in EMT and MAT [29, 42], our results suggest that they play distinct roles, with RhoA/RhoB inhibition and RhoC promotion of PCa cell migration and dissemination. Thus, the inhibition of RhoA/RhoB/ROCK signaling by itself induces mesenchymal cell invasion and tumoroid scattering (Fig. 4D, E), probably due to the stimulation of the PI3K/AKT/Rac1 pathway [40, 54]. Notably, combined ROCK and neddylation inhibition (Y27632 plus MLN) changed the morphology of disseminated cells from mesenchymal to amoeboid. As ROCK activity is involved in amoeboid migration, this suggests that Rho stimulation by MLN could overcome the effect of ROCK inhibitors. Otherwise, it is plausible that other Rho effectors, such as RhoC-specific FMNL2/3 [39, 42], mediate amoeboid invasion, as has been shown previously for FMNL2 [42]. RhoC is the only known GTPase essential for metastasis [55, 56] and is required for amoeboid migration [42, 43]. We show that MLN stimulates RhoC and induces its relocalization to the cell cortex and protrusions, which correlates with the increase in cell motility and tumoroid invasion (Fig. 5F, G; Supplementary Figure S2B). MLN also activates AKT1 and PRK3/PKN3 kinases (Fig. 2C; Supplementary Figure S5A), both of which are involved in the metastatic function of RhoC [26, 57]. Finally, RhoC depletion suppresses tumoroid growth and invasion (Fig. 4D, E), further supporting its prometastatic role.

Because either RhoC depletion or TJSR stimulation effectively blocks cancer cell growth and invasion, we suggest that the targeting of neddylation-dependent TJSR suppressors could be safer than NAE1 inhibition, as it would not stimulate prometastatic RhoC. Our deconvolution screening identified CUL2, DCNL4, and UBE2F as potential targets. Notably, the depletion of UBE2F and CUL2 suppresses PCa cell and tumoroid growth as efficiently as RhoC knockdown, suggesting that it is a promising therapeutic strategy. Plausibly, all three hits could form a functional cullin-RING ligase (CRL2): CUL2 was identified as a preferred cullin partner for DCNL4 [58], and UBE2F showed some promiscuity for cullin substrates [59]. However, the published data on their respective specificities argue against this possibility [59]. Because UBE2F is the only E2 enzyme capable of neddylating the CUL5/RBX2 complex, it is considered CRL5-specific. Thus, UBE2F has been shown to regulate cancer cell apoptosis and invasion through CRL5-dependent degradation of the proapoptotic protein NOXA and Bim [60, 61]. Nevertheless, our results suggest that UBE2F regulates TJSR independently of CRL5. The identification of the mechanisms underlying this regulation would help develop novel anticancer treatments that trigger TJSR. Stimulating TJSR could also be a way to increase the efficacy of MLN treatment. Thus, our drug screen identified 11 compounds that stimulated MLN-dependent TJSR by more than two-fold (Fig. 3B; Supplementary Figure S7; Supplementary Table S1). These drugs may serve as potential candidates for combination therapy with MLN in prostate cancer.

## Conclusions

Here, we uncover an important new function of the neddylation inhibitor pevonedistat (MLN4924) as a mechanotherapeutic. We found that tight junctions and mechanical stress induced by the inhibition of neddylation define its therapeutic outcome. Notably, the mechanical stress controls cancer cell survival and, in some cell types, can promote prometastatic behavior (Fig. 6D). We analyzed cellular pathways involved in this regulation and identified cancer cell vulnerabilities that help prevent adverse effects and improve the efficacy of neddylation inhibition. We also identified several drug candidates for use in combination therapy with pevonedistat.

## Supplementary Information

**Additional file 1.**

## Data Availability

The datasets supporting the conclusions of this article are included within the article and Supplementary Information.

## Abbreviations

C3E: *Clostridium botulinum* C3 exoenzyme
C-CPE: C-terminal fragment of *Clostridium perfringens* enterotoxin
Cldn4: Claudin 4
CNFy: *Yersinia pseudotuberculosis* Cytotoxic necrotizing factor
E-Cad: E-cadherin
EMT: Epithelial-to-mesenchymal transition
ItgB1: Integrin β1
MAT: Mesenchymal-to-amoeboid transition
MLN: MLN4924 (International Nonproprietary Name: Pevonedistat)
Ocln: Occludin
PCa: Prostate cancer
RNAi: RNA interference
TJ: Tight junction
TJSR: Tight junction stress response
