# Correction: Mechanical stress shapes the cancer cell response to neddylation inhibition

**DOI:** 10.1186/s13046-022-02371-9

**Published:** 2022-05-06

**Authors:** Frédérique Mittler, Patricia Obeïd, Vincent Haguet, Cédric Allier, Sophie Gerbaud, Anastasia V. Rulina, Xavier Gidrol, Maxim Y. Balakirev

**Affiliations:** 1 University Grenoble Alpes, CEA, INSERM, IRIG, Biomics, 38054 Grenoble, France; 2 University Grenoble Alpes, CEA, LETI, 38054 Grenoble, France; 3 University of Bergen, Bergen, Norway

**Correction to: J Exp Clin Cancer Res 41, 115 (2022)**

**https://doi.org/10.1186/s13046-022-02328-y**

Following publication of the original article [1], an error was identified in Fig. 1; specifically:

- Fig. 1b: Incorrect image used for Pulse-MLN cells (bottom right panel); the correct image is now used

The corrected figure is given here. The correction does not have any effect on the final conclusions of the paper. The original article has been corrected.

**Fig. 1:**
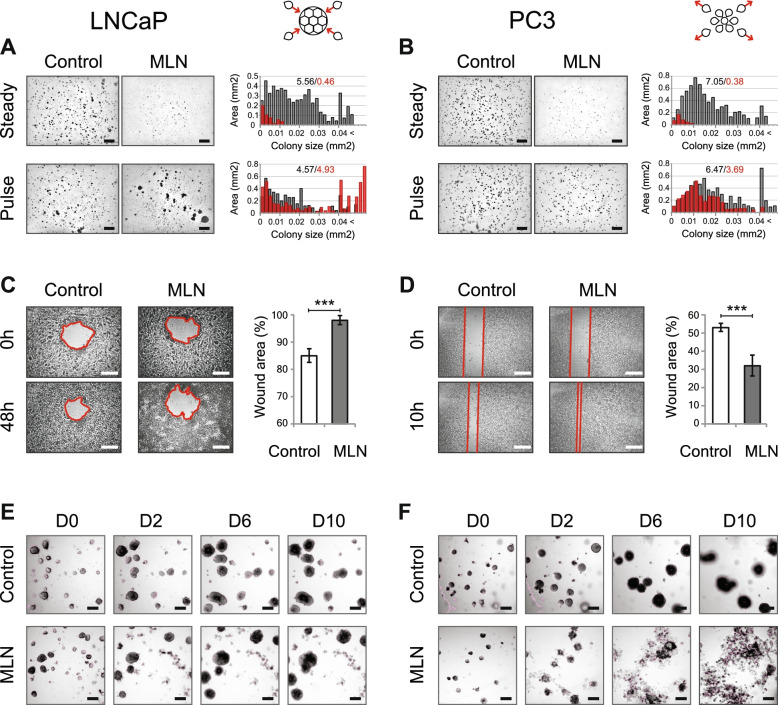
Neddylation inhibition induces distinct phenotypes in PCa cells. **A, B** Effect of 100 nM MLN on PCa colony growth in soft agar (“steady”). In the “pulse” regimen, the cells were pretreated with 100 nM MLN for 1 h before seeding (see also Supplementary Figure S1A). The histograms show the colony size distribution of control (black)- and MLN (red)-treated cells. The numbers indicate the total area occupied by the colonies of the given size. Scale bar = 1 mm. **C, D** Wound healing assay with LNCaP (**C**) and PC3 (**D**) cells (mean ±} S.D., *n* = 5 for LNCaP, *n* = 6 for PC3, ***-*p* < 0.001). 100 nM MLN was added just before monolayer scratching. Scale bar = 500 μm (**E, F**) Effect of 100 nM MLN on LNCaP (**E**) and PC3 (**F**) tumoroid growth over 10 days. Scale bar = 150 μm. In all experiments with MLNs, DMSO was used as a vehicle control

## References

[1] Mittler F, Obeïd P, Haguet V (2022). Mechanical stress shapes the cancer cell response to neddylation inhibition. J Exp Clin Cancer Res.

