# Supplementary material for: Mechanical stress shapes the cancer cell response to neddylation inhibition
Source: J Exp Clin Cancer Res. 2022 Mar 30;41:115. doi: 10.1186/s13046-022-02328-y (PMC8966269; doi:10.1186/s13046-022-02328-y)
Supplement: Supplementary file 1 — Additional file 1. [file 13046_2022_2328_MOESM1_ESM.pdf]

SUPPLEMENTARY INFORMATION

Mechanical stress shapes the cancer cell response to neddylation inhibition

Frédérique Mittler[1], Patricia Obeïd[1], Vincent Haguet[1], Cédric Allier[2], Sophie Gerbaud[1], Anastasia V. Rulina[1,3], Xavier Gidrol[1], and Maxim Y. Balakirev[1*]

[1]Univ. Grenoble Alpes, CEA, INSERM, IRIG, Biomics, 38054 Grenoble, France.
[2]IUniv. Grenoble Alpes, CEA, LETI, 38054 Grenoble, France.
[3]University of Bergen, Bergen, Norway.
*Corresponding author, maxim.balakirev@cea.fr

RUNNING TITLE: Drug-induced mechanical stress in prostate cancer cells

TABLE OF CONTENTS

Pages

Supplementary Materials & Methods........ 2-10

Supplementary Figures S1-S14........ 11-24

Supplementary Tables S1-S4........ 25-28

Supplementary References........ 29

Supplementary Materials and Methods

Materials

Falcon® 96 Well Clear Round Bottom Non-Treated Assay plates (NTA plates, Corning #353910) and White 96 Well Transparent Bottom CELLSTAR® plates (white plates, Greiner Bio-One #655088) were purchased from Dominique Dutscher (Grenoble, France). Universal polystyrene lids (Thermo Fisher Scientific #5500), Dulbecco's modified Eagle's medium (DMEM, Gibco #41966), and Roswell Park Memorial Institute (RPMI1640, Gibco #61870) medium, Dulbecco's Phosphate-Buffered Saline, no calcium, no magnesium (PBS, Gibco #14190), 0.05% Trypsin–EDTA (Gibco #25300054), 10 kU/mL Penicillin-Streptomycin (Gibco #15140122), CellEvent Caspase-3/7 Green Reagent (Invitrogen #C10423) were obtained from Thermo Fisher Scientific (Courtaboeuf, France). Fetal bovine serum (FBS, PAN Biotech #P30-3302) was purchased from Dominique Dutscher (Grenoble, France). ViaLight™ Plus Cell Proliferation and Cytotoxicity BioAssay Kit (Lonza #LT07-121) was obtained from Lonza (Basel, Switzerland). See Supplementary Tables S1-S3 for Drugs, Antibodies, and siRNAs.

Cell culture

The LNCaP (androgen receptor-positive, androgen-dependent, p53-wt, PTEN-mut; ATCC #1740), VCaP (androgen receptor-positive, androgen-sensitive, containing TMPRSS-ERG translocation, p53-R248W, PTEN-wt; ATCC #CRL-2876), and PC3 (androgen receptor-negative, androgen-independent, p53-null, PTEN-null; ATCC #CRL-1435) cell lines were purchased from the American Type Culture Collection (ATCC). Cell lines were tested on a semester basis for AR, PSA, SLC45A3 and ERG by immunoblotting and quantitative RT-PCR. VCaP cells were cultured in DMEM containing 10% FBS and 1% Penicillin-Streptomycin (complete growth medium). PC3 and LNCaP cells were cultured in RPMI1640 with the same supplements. The cells were grown in an incubator at 37°C, 5% CO2, and 95% humidity. For different passages, the cells were washed twice with PBS followed by the addition of Trypsin-EDTA and incubation for 3-10 minutes depending on the cell line. Subculture was done depending on the density of the cells. Usually for VCaP cells, subculture was done once per week, with dilution to 1/2; LNCaP and PC3 were divided twice per week, with dilution to 1/5. Charcoal stripped serum was prepared as previously described [1].

Soft agar colony formation assay

For soft agar colony formation assays, a warm (52°C) 1:1 mixture of 1% agarose solution (Thermo Fisher Scientific #16500100) and DMEM/ Glutamax/20% FBS medium was poured into a 6-well plate (1ml per well) and allowed to solidify. In parallel, cells were detached with Trypsin/EDTA, neutralized with medium containing 10% FBS and counted using Trypan staining with an automatic cell counter EVE™ (NanoEnTek). The cells were then diluted with DMEM/Glutamax/20% FBS medium to obtain a concentration of 2000 cells/ml. Next, the cell suspension was mixed in 1:1 ratio with a warm (42°C) 0,7% low-melting-point agarose solution (Thermo Fisher Scientific #16520050) and layered over the preformed agarose layer in a 6-well plate (1ml per well). The cell-agarose layer was kept at room temperature until solidification. After that, 2 ml of DMEM/ Glutamax/20% FBS medium with or without 100 nM MLN was added. For "pulse" regimen, the cells were pre-treated with 100 nM MLN for 1 hour in suspension before mixing with the agarose solution. The plates were kept in an incubator (37°C, 5%

CO_2, 95% humidity) until colonies were formed, and stained with Nitro Blue Tetrazolium (NBT, Sigma-Aldrich). The images were acquired with ChemiTouch (Bio-Rad) and analyzed using Fiji software.

Wound healing assay

Cells were seeded at 40,000 cells/200µl/well into 96-well Black/Clear Flat Bottom Imaging plates (Falcon #353219) and grown until confluence for ~ 4 days. The medium was changed for the fresh medium containing different concentrations of MLN with or without 200 µg/ml C-CPE. Cell monolayers were scratched using a wound replicator equipped with 96 stainless steel pins (V&P Scientific, San Diego, California). The treatments were performed in quintuplicate (LNCaP) or sextuplicate (PC3). The imaging was performed within the cell culture incubator on in-line holographic microscope equipped with 96 quasi-coherent light sources and image sensors. This in-house device allows parallel filming of individual wells in 96-well microtiter plate with a large field of view (2.8×2.1 mm^2) [2,3]. The captured images were quantified using ImageJ software.

Lens-free microscopy and holographic reconstruction

High-content holographic imaging of cultured cells was performed within the cell culture incubator using lens-free Cytonote microscope (Iprasense, Montpellier, France). Illumination is provided by a red LED (647 nm, FWHM 13 nm). The light passes through a 150 µm pinhole at a distance of approximately 5 cm from the sample. The CMOS sensor is in contact with the cell culture recipient at a distance of ~1.5 mm. Compared to conventional optical methods, lens-free microscopy provides a unique way to track thousands of live cells in real time and with a large field of view (35 mm^2) [4]. In the absence of optics, the sample image is obtained by a reconstruction algorithm. To analyze cell dynamics Trackmate algorithm, an open source Fiji plugin for the automated tracking of single particles [5], was used. The images were acquired every 20 min during several days. High-content analysis of the data provides detailed quantification of each of thousands of single objects with such parameters as surface area, aspect ratio, dry mass, and velocity.

Spheroid culture

Spheroid culture and analysis were performed as previously described [6]. Cells were suspended in the appropriate complete growth medium and filtered when required through a 40 µm cell strainer to ensure that the cell suspension was homogeneous (single cell). After the cell density was counted using an automatic cell counter EVE™ (NanoEnTek) and adjusted with a growth medium, the cells were distributed into 96-well Non-Treated Assay U-bottom plates (Falcon #353910) to obtain 500-2000 cells per well (depending on the cell line) in 200 µl growth medium. The plates were covered with the universal polystyrene lids and kept in an incubator (37°C, 5% CO_2, 95% humidity) for at least 2 days before imaging. To analyze the effects of drugs on spheroid morphology and viability, 100 µL of spheroid medium was removed from each well and replaced by 100 µL of drug solution. The plates were kept in an incubator, and the image acquisition was performed once a day. When analyzing apoptosis, CellEvent was added to each of the drug solutions to obtain a 2 µM concentration.

The analysis of spheroid assembly was conducted immediately after the cell suspension distribution using the video microscope equipped with a cell culture chamber (Zeiss Axiovert 200M). The

acquisitions were made every 60 minutes during 4 days. Typically, 10 spheroids per condition were monitored.

Tumoroid culture in Matrigel

Tumoroids were grown from a single cell suspension in Matrigel (Corning #356231). Matrigel was diluted with cold PBS to 7 mg/ml and 50 µl aliquots were distributed into 96-well Black/Clear Flat Bottom Imaging plates (Falcon #353219). The plates were kept in a cell incubator at 37°C for 45 min to polymerize Matrigel. Next, 500 cells in 100 µl of single cell suspension (see "Spheroid culture") were distributed into each well and allowed to adhere to the Matrigel bed for 30 min at 37°C before addition of 100 µl of complete growth medium with 8% Matrigel. The plates were kept in an incubator (37°C, 5% CO2, 95% humidity) for at least 4 days before imaging.

To analyze the effects of drugs on tumoroid morphology and viability, 100 µL of the medium was removed from each well and replaced by 100 µL of drug solution in the same medium. To analyze the effects of protein depletions, cells were transfected with siRNAs for 24 h (see below) collected and used for tumoroid culture as describe above. The plates were kept in an incubator, and the image acquisition was performed once a day. The treatments were performed at least in triplicates.

Analysis of tumoroid morphology was performed once a day on the CellInsight NXT High Content Screening Platform (Thermo Scientific) using 4x and 10x objectives. The acquisition parameters were set using the instrument software HCS Studio 6.5.0. The bright field channel was used for autofocus, and the exposure time was set within the 25%-35% saturation range according to the HCS Studio recommendations. When required, the bright field images were imported as "Channel 1- Object" and used to generate the object masks by imposing the size range for the counted objects. The acquisition of "four fields of view per well" was used to visualize the entire well with 4x objective. For each condition, a composite image was generated by Z-stacking (eleven Z-steps of 20 µm). To analyze tumoroid size and invasion into the Matrigel, maximum intensity projection images were quantified using in-lab ImageJ macro.

ATP-based viability assay

Cell metabolism was analyzed by measuring ATP content using ViaLight™ Plus Cell Proliferation and Cytotoxicity BioAssay Kit according to the manufacturer's protocol (Lonza #LT07-121). In short, cells were seeded in white plates with transparent bottom suitable for luminescence assays (Grenier #655088). The treatments were performed on the next day after cell seeding. At indicated times, 50 µL of cell lysis reagent was added to the cells directly into culture medium for 10 minutes, followed by addition of 100 µL of room temperature ATP monitoring reagent plus (AMR-plus) for 5 minutes. Blank solution was prepared by adding cell lysis reagent and AMR-plus to complete growth medium. Luminescence was measured using GloMax®-Multi Detection System (Promega) with a 1 sec integration time.

To analyze spheroid and tumoroid viability the same protocol was applied with some modifications [6]. Thus, 100 µL of medium was removed from each well and 50 µL of cell lysis reagent was added. The plate was agitated on an orbital shaker for 20 min at room temperature. Then, 100 µL of room temperature AMR-plus was added to each well and the plate was incubated for 2 min at room temperature before measuring luminescence.

Fluorescence microscopy and flow cytometry

Immunofluorescence microscopy was performed in 96-well Black/Clear Flat Bottom plates (Falcon #353219) using Axioimager Z1 Apotome fluorescence microscope (Zeiss). All procedures were performed at room temperature. Culture medium was removed and the cells were fixed in 100 µl 4% paraformaldehyde (PFA) in PBS for 15 min. The cells were washed twice with PBS supplemented with Ca2+ and Mg2+ (PBS++, Sigma, P4417), permeabilized with 0.2% Tween in PBS for 5 min, washed twice with 0.5% BSA in PBS++, incubated for 30 min in 100 µL of 3% BSA in PBS++, and washed twice with the same solution. The incubation with a primary antibody was performed in 80 µL of 0.5% BSA in PBS++ for 1 h followed by two washes 3% BSA in PBS++. The incubation with a fluorophore-labeled secondary antibody and phalloidin (to label actin filaments) was performed similarly. Finally, cell nuclei were stained with 5 µM Hoechst 33342 dye solution in 100 µL PBS++ for 5 min, and the cells were washed twice with PBS++, covered with 100 µL of 50% glycerol in PBS++ and stored at 4°C until analysis by fluorescence microscopy. The antibodies used for immunofluorescence microscopy are listed in Supplementary Table S2.

To analyze cell morphology, cells were labeled with HCS CellMask™ Green Stain according to the manufacturer's protocol (Thermo Fisher Scientific # H32714). The imaging was performed on the automated CellInsight NXT High Content Screening Platform (Thermo Scientific) using 10x and 20x objectives. The images were analyzed using the instrument software HCS Studio 6.5.0-“Morphology.V4”. The cell “Area”, aspect ratio “LWR” (Length to Width Ratio, representing cell asymmetry) and “Shape P2A” were chosen as the most informative morphometric parameters. Shape P2A = (Perimeter)2/ 4π x (Area) represents the cell roundness and is equal to 1 for a perfect circle; for less spherical objects, P2A becomes larger than 1. Note that for the manually acquired microscope images we used in-lab ImageJ macro that calculates an inverse roundness parameter, “Circularity”, which is equal to 1/P2A. Thousands of individual cells were analyzed per replicate. A table of all calculated features was generated as a .csv file using the “Cellomics-View” application. The data were imported into “R” statistical software to create a fully annotated .csv data file by fusing with the “Experimental Design” table. Statistical analyses have been performed using the statistical software R (http://www.r-project.org/). Box and whiskers plots represent the distribution of the data, with the box delimiting the central half of the data (from first to third quartiles); the segment is the median of the data. The whiskers delimit the rest of the data if its length does not exceed 1.5 times the size of the box, other data points are indicated by circles. P-values are calculated using the two-sided Wilcoxon rank-based test.

To measure apoptosis, cells were cultured in the presence of 2 µM of CellEvent™ Caspase-3/7 Green Detection Reagent (Thermo Fisher Scientific #C10423). The cells were fixed with PFA stained with 5 µM Hoechst 33342, and imaged on CellInsight as describe above. The images were analyzed by using HCS Studio 6.5.0- “Cell Health Profiling” program. Automatic segmentation of the nuclei was performed with the Hoechst channel, and used to measure the nuclear fluorescence intensity of CellEvent reagent. The cells with CellEvent signal above a certain threshold were considered apoptotic. The mean, median, standard deviation (S.D.), and quartile values were calculated with “R” software.

Analysis of C-CPE binding to PCa cells was performed both with live and fixed cells. LNCaP and VCaP cells were grown to 70%-80% confluence and treated with different concentrations of MLN for 24 h. The medium was changed for the fresh medium containing 10 µg/ml of Cy3-labeled C-CPE and live imaging was started after 1 h of incubation. For endpoint analysis, the medium was removed; the cells were fixed in 4% PFA in PBS for 15 min, washed with PBS and imaged on the microscope. For FACS analysis, ~5 x 105 cells were incubated with Cy3-labeled C-CPE (70 µg/ml) in 100 µl suspension for 1 h

at room temperature. Then, the cells were washed with PBS and analyzed on the BD LSR II flow cytometer (BD Biosciences).

Time-lapse confocal microscopy of actin dynamics

To study actin dynamics, LNCaP cells were transduced with CellLight® Actin-RFP, BacMam 2.0 reagent according to the manufacturer's protocol (Thermo Fisher Scientific #C10502). After 24 h, the medium was replaced with the medium containing drugs and the imaging was performed on a confocal spinning-disc system (EclipseTi-E Nikon inverted microscope equipped with a CSUX1-A1 Yokogawa confocal head, an Evolve EMCCD camera from Roper Scientific, Princeton Instruments). The acquisition was performed from nine fields per condition every 10 min for 20 h, and maximum intensity projection images were processed using in-lab ImageJ macro to quantify F-actin.

Recombinant proteins (C-CPE [7], C3E [8], GST-CNFy [9], GST-RBD [10], GST-PAK-CRIB [11])

The recombinant proteins were produced according to standard protocols. The plasmids were transformed into BL21 Star (DE3) Chemically Competent *E. coli* cells (Thermo Fisher Scientific #C602003) and the protein expression was induced by 100 µM isopropyl β-D-1-thiogalactopyranoside (IPTG) for 6 h at room temperature. The cells were collected by centrifugation and lysed by sonication in cell lysis buffer (CLB, 20 mM HEPES pH 7.5, 150 mM NaCl, 10% glycerol) supplemented with 3 mM MgCl2, 1 mM dithiothreitol (DTT), 1% Triton X-100 (Tx-100), cOmplete™ EDTA-free Protease Inhibitor Cocktail (Roche #11836170001), 1 mM phenylmethylsulfonyl fluoride (PMSF), and 10 µg/ml RNAse A. The lysates were clarified by centrifugation at 25000g for 30 min, filtered through 0.2 µm filter and the proteins were purified by affinity chromatography on HisPur Cobalt resin (Thermo Fisher Scientific #89964) for his-tagged C-CPE and C3E proteins or on Glutathione Sepharose® 4 Fast Flow resin (Cytiva #17513201) for GST-CNFy, GST-RBD and GST-PAK-CRIB proteins. C-CPE protein was eluted with 250 mM imidazole in CLB, dialyzed against 10% glycerol in PBS and stored frozen at -80°C. C3E protein was eluted with 250 mM imidazole, 1 mM DTT in CLB, dialyzed against CLB and stored frozen at -80°C. GST-CNFy, GST-RBD and GST-PAK-CRIB proteins were eluted with 10 mM GSH, 1 mM DTT, and 1% Tx-100 in CLB, dialyzed against CLB and stored frozen at -80°C.

C-CPE protein was fluorescently labeled with Cy3 NHS-ester (SIGMA #GEPA13101). The freshly prepared 20 µl of 1 M NaHCO3 was added to 180 µl of the protein (1.3 mg/ml in PBS-10% glycerol). Then, 0.5 µl of 100 mM Cy3 NHS-ester Cy3 solution in DMSO was added and the mixture was vortexed at 350 rpm for 1 h at 23°C. The reaction was quenched with 10 µl of 1 M ethanolamine and the mixture was purified on 2 ml Zeba Desalting Column (7K MWCO, Thermo Fisher Scientific #89890) according to the manufacturer's instructions. The protein was eluted in 210 µl of PBS-10% glycerol and dialyzed overnight at 4°C against the same solution. The dialyzed solution was centrifuged at 20000 g for 30 min at 4°C, and the supernatant was aliquoted and stored frozen at -80°C. The yield was ~200 µl of 0.35 mg/ml C-CPE-Cy3 with labeling ratio Cy3/Protein = 2.3 (based on Nanodrop absorbance measurements using C-CPE and Cy3 molar extinction coefficients).

Western blotting

For western blot analysis, cells were grown in 6-well Clear Flat Bottom TC-treated Cell Culture plates (Falcon #353046). The cells were washed twice with PBS and collected using Trypsin-EDTA treatment.

Cellular proteins were extracted using RIPA lysis buffer (Sigma #R0278) supplemented with cOmplete™ protease inhibitor cocktail (PIC, Roche #11836153001), 10 mM *ortho*-phenanthroline, 30 mM N-ethylmaleimide, 5 mM sodium ortho-vanadate, and 5 mM sodium fluoride (RIPA++). Alternatively, for membrane proteins sensitive to Trypsin-EDTA (i.e. ItgB1), the cells were treated directly in the plate with 100 µl RIPA++, frozen at -20°C overnight, thawed and processed as below. After quantification with a BCA protein assay kit (Pierce #23225), an equal amount of protein (typically 20 µg per sample) was run on NuPAGE Novex Bis-Tris Gel (Thermo Fisher Scientific #NP0322BOX, #EC60252BOX, #NP0323BOX) in MES buffer and then transferred onto the nitrocellulose membrane (Amersham™ Protran®, Cytiva #10600001). The membranes were blocked in 5% nonfat milk/TBST for 40 min at 37°C, incubated with primary antibodies in 5% nonfat milk/TBST for 1 hour at RT or overnight at 4°C. This step was followed by incubation with secondary HRP-conjugated antibodies. Detection was performed on ChemiTouch instrument (Bio-Rad) using an appropriate chemiluminescent reagent (Plus-ECL, Perkin Elmer #NEL105001EA; ECL Prime, Cytiva #RPN2232; SuperSignal West Femto, Thermo Fisher Scientific #34094, depending on the signal intensity). The western blot images were quantified using ImageJ software. The antibodies used for western blotting are listed in Supplementary Table S2.

Small GTPase assay

The analysis of small GTPases was performed as described [12,13] with some modifications. Selective capture of GTP-bound forms of GTPases was performed on the beads coated with the affinity ligands: Rhotekin Rho Binding Domain (RBD) and the Cdc42- and Rac-Interactive Binding motif (CRIB). The beads were prepared by incubating GST-tagged ligands with Glutathione Sepharose® 4 Fast Flow resin for 1 h at 4°C (1 mg of recombinant protein in CLB per 100 µl of beads, Cytiva #17513201). The beads were washed with cold CLB and equilibrated with the corresponding GTPase Lysis Buffer: RBD-LB (RIPA complemented with 350 mM NaCl, 10 mM MgCl2, 1 mM PMSF, and cOmplete™ PIC) or CRIB-LB (50 mM Tris-HCl, pH 7.5, 200 mM NaCl, 5 mM MgCl2, 1 mM DTT, 1% NP-40, 10% glycerol, 1 mM PMSF, and cOmplete™ PIC). The beads were aliquoted (20 µl per 1.5 ml eppendorf tube) and kept on ice until use.

For GTPase assay, cells were grown in 6-well plates with two (Rho) or four (Cdc42 and Rac) wells per assay condition/ replicate. Cells were washed twice with ice-cold PBS and lysed on ice in RBD-LB or in CRIB-LB (200 µl/well). The lysates corresponding to the same condition/ replicate were pooled, cleared by centrifugation at 20800 g for 10 minutes at 4°C, applied onto the beads and rotated for 60 minutes at 4°C. An aliquot was saved to assess protein concentration and total GTPase level. Beads were washed three times with ice-cold lysis buffer, the bound GTPases were eluted in 50 µl of 2x Laemmli buffer and analyzed by western blotting using specific antibodies.

Immunoprecipitation

Immunoprecipitations were performed essentially as described [14]. LNCaP cells were grown in T75 flask, harvested and lysed at 4°C with 750 µl of extraction buffer (PBS containing 1% Tx-100, 10 mM sodium ortho-vanadate, 10 mM sodium fluoride, 1 mM PMSF, and cOmplete™ PIC). The lysate was cleared by centrifugation at 13,000 g for 15 minutes at 4°C, and applied onto 15 µl of NHS Mag Sepharose beads (Cytiva #28951380) covalently coupled to ZO-1 Antibody (Thermo Fisher Scientific #61-7300) or to Rabbit IgG Isotype Control (Thermo Fisher Scientific #02-6102) according to the manufacturer's instructions. After rotating for 2 h at 4°C, the beads were washed twice with extraction buffer and once with PBS. Bound proteins were eluted in 50 µl of 2x Laemmli buffer and analyzed by western blotting.

Isolation of ubiquitylated proteins on multiDSK resin

Enrichment of ubiquitylated proteins on multiDSK resin was performed as described[15] with some modifications. The plasmid coding for GST tagged multiDSK construct [15] was transformed into BL21 Star (DE3) Chemically Competent E. coli cells and protein expression was induced by 400 µM isopropyl β-D-1-thiogalactopyranoside (IPTG) for 6 h at room temperature. The cells from 800 ml culture were collected by centrifugation and lysed by sonication in 20 ml of cell lysis buffer (STE, 10 mM Tris pH 8; 1 mM EDTA, 100 mM NaCl) supplemented with cOmplete™ EDTA-free Protease Inhibitor Cocktail, 1 mM phenylmethylsulfonyl fluoride (PMSF), 10 µg/ml RNAse A, and 1.5% N-lauryl sarcosine. Triton X-100 was added to the lysate to a final concentration of 3% to mask the sarcosine. The lysate was clarified by centrifugation at 40000g for 1 h, filtered through 0.2 µm filter and mixed with 0.8 ml of pre-equilibrated Glutathione Sepharose® 4 Fast Flow resin. The bead suspension was rotated for 4 hours at 4°C and loaded into the column. The beads were washed thoroughly in STE buffer containing 500 mM NaCl and 0.1% Triton X-100, followed by a 50 mM NaCl wash in the same buffer, and finally STE with 10% glycerol. The beads were resuspended in STE- 10% glycerol and used in pulldown assays the same day. MultiDSK beads can also be aliquoted and stored frozen at -80°C.

For multiDSK pulldown assay, LNCaP cells were grown in T25 flasks. The cells treated at 70% confluency with 50 nM MLN or DMSO as a vehicle control for 20 h and then with 500 nM bortezomib (Btz) or DMSO for 4 h. The cells with were harvested by trypsinization and washed once with PBS. The cell pellet from one flask was lysed in 600 µl of ice-cold cell lysis buffer (CLB, TBS containing 5mM EDTA, 1% NP 40, Protease Cocktail Inhibitors, 30 mM NEM, and 5mM OPA). The lysate was clarified by centrifugation for 20 min at 20000 g (4°C). The supernatant was added to 30 µl of pre-equilibrated multiDSK beads and rotated 4 h at 4°C. The beads were collected by centrifugation and washed once with 1 ml of CLB and three times with 1ml of TBS-5mM EDTA. The proteins were eluted by heating the beads in 100 µl 1,5x Laemmli buffer and analyzed by western blotting.

Analysis of Rho modifications using his6-tagged Rho proteins

The DNA sequences coding for RhoA, RhoB, and RhoC have been amplified by RT-PCR from LNCaP cells. The PCR reactions were performed with two sets of primers to introduce N-terminal-his6 coding sequences and restriction sites: KpnI-XhoI (RhoA), NheI-BglII (RhoB), and NheI-XhoI (RhoC). The PCR primers were as follows:

hRhoA1 : CATCATCAT GGA ATGGCTGCCATCCGGAAGAAA
hRhoA2 : TAT GGTACC ATG CATCATCATCATCATCAT GGA ATGGCTGCC
hRhoAr : ATT CTCGAG TCACAAGACAAGGCACCCAG
hRhoB1 : CATCATCAT GGA ATGGCGGCCATCCGCAAGA
hRhoB2 : TAT GCTAGC ATG CATCATCATCATCATCAT GGA ATGGCGGCC
hRhoBr : ATA AGATCT CTCGAG TCATAGCACCTTGCAG
hRhoC1 : CATCATCAT GGA ATGGCTGCAATCCGAAAGAAGC
hRhoC2 : TAT GCTAGC ATG CATCATCATCATCATCAT GGA ATGGCTGCAAT
hRhoCr : ATT CTCGAG TCAGAGAATGGGACAGCCCC

The genes were cloned into the corresponding restriction sites in pcDNA3.1(+) vector and sequenced. For protein analysis, LNCaP cells were grown in 6-well plates until they reached 70% confluence. The cells were transfected with 2.5 µg of his6-Rho plasmid per well and 7.5 µl/ well of Lipofectamine® 2000 Transfection Reagent (Thermo Fisher Scientific #11668019) according to the manufacturer's protocol. On the next day, the cells were treated with 50 nM MLN or DMSO as a vehicle control for 20 h and

then with 500 nM bortezomib (Btz) or 5 nM bafilomycin A (Baf A) for 4 h. Three wells per experimental condition were used for his6-Rho purification (one well for expression control, or "load", Figure S8C,D). The his6-tagged Rho proteins were isolated in denaturing conditions using immobilized metal affinity chromatography (IMAC). The cells were lysed directly in the wells using 200 µl/well of denaturing lysis buffer (dLB, PBS, 6 M guanidium-HCl, 0.1 % Triton X-100, 1% NP 40, Protease Cocktail Inhibitors, 30 mM NEM). The lysates for each condition were pooled (~600 µl), sonicated for 10 min in a water bath sonicator, and centrifuged for 30 min at 20000 g. The clarified lysate were then added to 30 µl of pre-equilibrated Ni-NTA Superflow resin (QIAGEN) and rotated 4 h at room temperature. The beads were collected by centrifugation and washed three times with 1 ml of dLB and three times with 1ml of PBS-0.1 % Triton X-100. The proteins were eluted in 60 µL of 2× laemmli/ 200 mM imidazole and analyzed by western blotting.

Cellular fractionation

Crude subcellular fractionation was performed essentially as described [16]. Briefly, cells were washed with ice-cold PBS, resuspended (2× 107cells/ml) in NP40 lysis buffer (50 mM HEPES pH 7.5, 150 mM NaCl, 1% NP40, 1 mM PMSF, and cOmplete™ PIC) and incubated on ice for 30 min. The lysate was centrifuged at 7,000 g for 10 minutes at 4°C to separate the nuclei (pellet) from the cytosol and membranous organelles (supernatant). Nuclear proteins were extracted by boiling the pellet in Laemmli buffer and analyzed along with the supernatant fractions by western blotting.

Luciferase reporter assay

Cells were grown in white 96-well plates with transparent bottom suitable for luminescence assays (Grenier #655088) until they reached 70%-80% confluence. Next, cells were co-transfected in triplicates with CLDN4 Firefly luciferase reporter [17] and a constitutively-active Renilla luciferase reference vector (1:10 w/w ratio, 200 ng of total DNA per well) and with 0.5 µl/ well Lipofectamine® 2000 Transfection Reagent (Thermo Fisher Scientific #11668019) according to the manufacturer's protocol. The cells were treated with MLN on the day after transfection and analyzed after 24 hours. Luciferase measurements were performed using the Dual-Luciferase Reporter Assay (Promega #E1910), according to the manufacturer's instructions using GloMax®-Multi Detection System (Promega). The ratio of Firefly- to Renilla-luciferase activities was calculated. All values were presented as means ± SD.

RNA extraction, RT-PCR, and qPCR

RNA was extracted with an RNeasy Mini Kit (QIAGEN #74104). 1.5 µg RNA was reverse-transcribed in a total volume of 20 µl using a SuperScript® VILO cDNA Synthesis Kit (Thermo Fisher Scientific #11754050) with random primers according to the manufacturer's protocol. Reverse transcription reactions were diluted to 200 µl of distilled water and further used in concentrations of 2.5 µl per reaction of quantitative PCR (qPCR). qPCR was carried out with a Platinum Quantitative PCR SuperMIX-UDG Kit (Thermo Fisher Scientific #11730-017) using a StepOnePlus Real-Time PCR system (Applied Biosystems #4376600). All experiments were run in triplicates, and the results were normalized to GAPDH mRNA expression. qPCR primers were as follows: forward primer for *CLDN4*, 5′-TGCCTGGAGGATGAAAGCG-3′; reverse primer for *CLDN4*, 5′-GAAGTCTTGGATGATGTTGTGGG-3′; forward primer for *OCLN*, 5′-GCCGGTTCCTGAAGTGGTT-3′; reverse primer for *OCLN*, 5′-

CGAGGCTGCCTGAAGTCATC-3′; forward primer for *GAPDH*, 5′-TCCCTGCCACACTCAGTCC-3′; reverse primer for *GAPDH*, 5′-GAGCACAGGGTACTTTATTGATGG-3′.

Drug screen

The effect of drugs on Clnd4 expression was analyzed by western blotting. The treatments were performed at ~70% of cell confluence in 6-well plates. The drugs were diluted in cell medium from DMSO stocks (0.5% DMSO in the final drug solution with or without 100 nM MLN). Pure 0.5% DMSO was used as a control condition. After 24 h, the cells were processed as described above and the western blot images were quantified using ImageJ software. A list of the drugs is given in Supplementary Table S1.

siRNA transfection

Cells were transfected at ~70% of cell confluence in 6-well plates using 10 nM siRNA and Lipofectamine® RNAiMAX Transfection Reagent according to the manufacturer's instructions (Thermo Fisher Scientific #13778). For Clnd4 expression analysis, the cells were processed for western blotting 48 h post transfection. Both siGENOME® SMARTpool and individual siRNAs were used. A list of siRNAs is given in Supplementary Table S3.

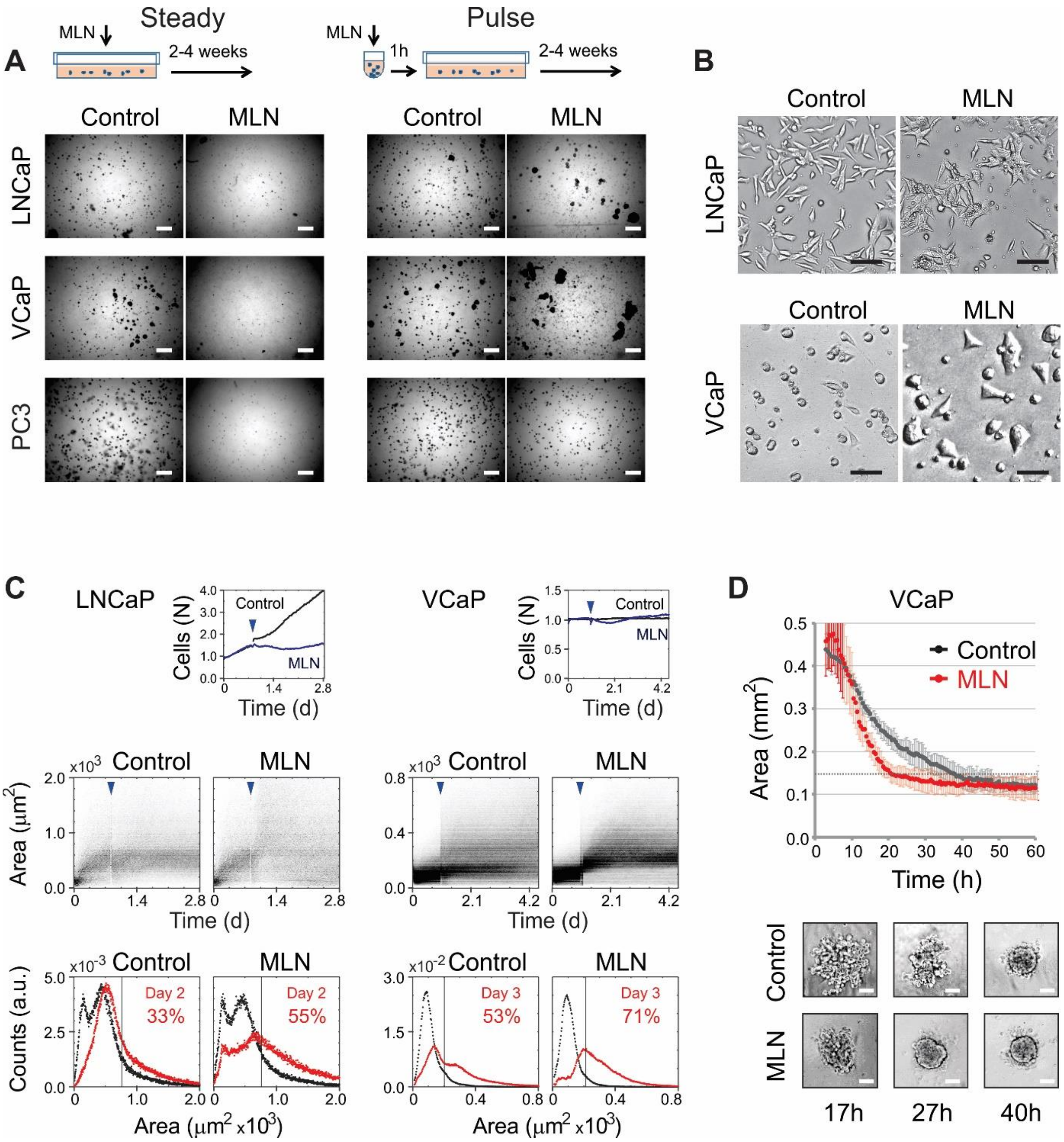

Supplementary Figure S1. Effect of MLN on PCa cell phenotype. (A) "Steady" and "pulse" regimens to analyze the effect of MLN treatment on PCa colony growth in soft agar. The concentration of MLN was 100 nM, and DMSO was used as a vehicle control. Scale bar = 1 mm. (B) PCa cell clustering upon treatment with 100 nM MLN for 24 hours. Scale bar = 100 µm. (C) Quantitative analysis of the effects of MLN on LNCaP and VCaP cells using lens-free video microscopy. The histograms on the top show the change in cell count within the observation field normalized to the initial number (1028 LNCaP cells and 950 VCaP cells, respectively). Blue arrowheads indicate the addition of 100 nM MLN or DMSO (control). The middle graphs show the size distribution over time, where each point corresponds to one cell or small cell cluster. The bottom diagrams show the size distribution just before the drug addition (black dots) and on the day 2 (LNCaP) or day 3 (VCaP) of the experiment (red dots). The percentage of the objects with the size above the threshold is given within the graphs. (D) The change in projected area of VCaP spheroids during the assembly in U-bottomed plates (mean ± S.D., n=6). The concentration of MLN was 100 nM, and DMSO was used as a vehicle control. Representative images are shown below. Scale bar = 200 µm.

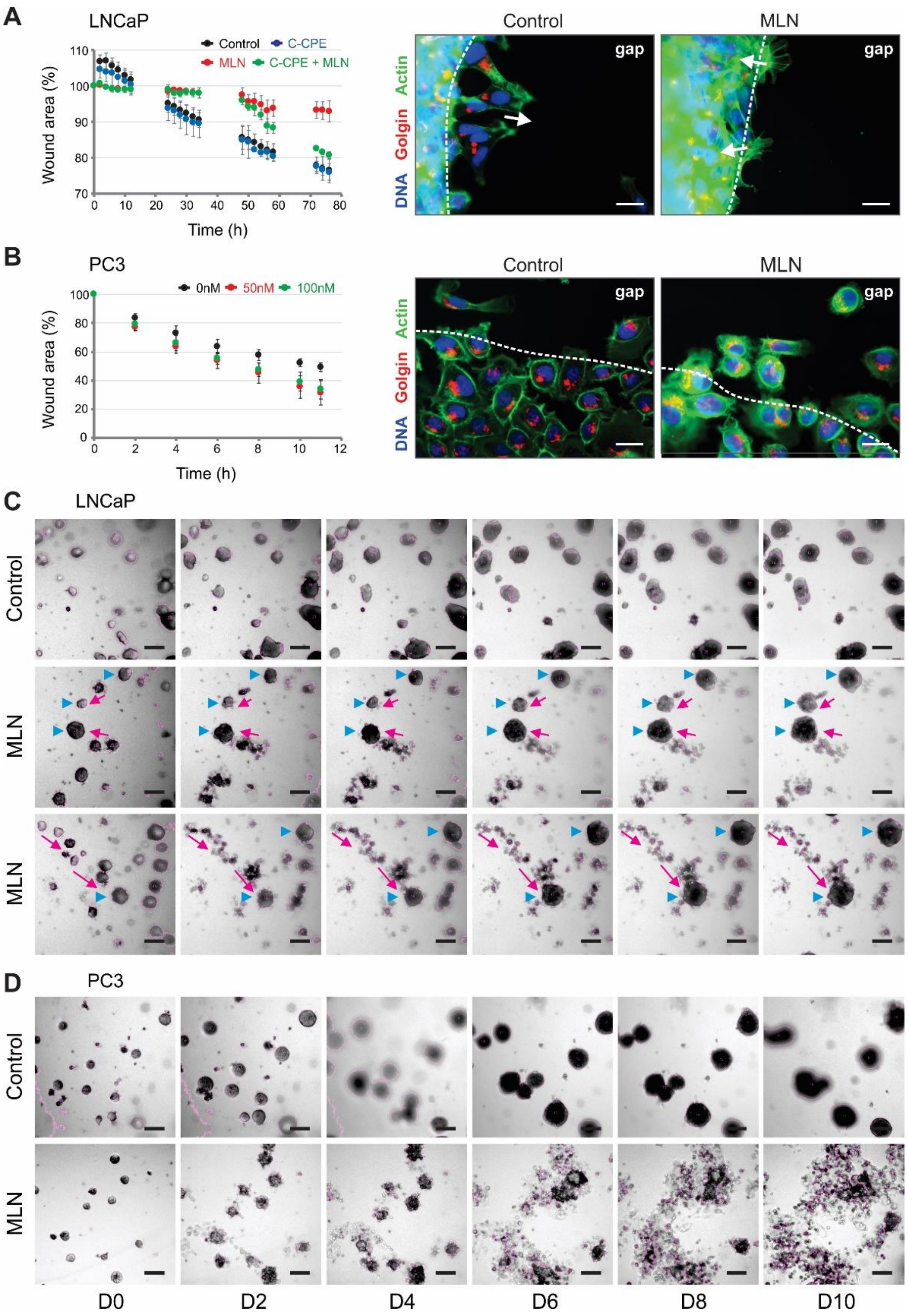

Supplementary Figure S2. Effect of MLN on PCa cell phenotype. (A) 100 nM MLN inhibits LNCaP cell migration in a wound healing assay. The addition of 200 µg/ml C-CPE counteracts the effect of MLN (mean ± S.D., n=5). Representative images on the right show the directions of cell projections (white arrows) relative to the wound gap. Scale bar = 25 µm. (B) Effect of MLN on PC3 cell migration in a wound healing assay (mean ± S.D., n=6). Representative images on the right are shown for 100 nM MLN. Scale bar = 25 µm. (C) Effect of 100 nM MLN and DMSO (control) on LNCaP tumoroid growth over 10 days. The blue arrowheads indicate resistant clones which are attracting and swallowing smaller tumoroids and their remnants (magenta arrows). Scale bar = 150 µm. (D) Effect of 100 nM MLN on PC3 tumoroid growth over 10 days. Scale bar = 150 µm. In all experiments with MLN, DMSO was used as a vehicle control.

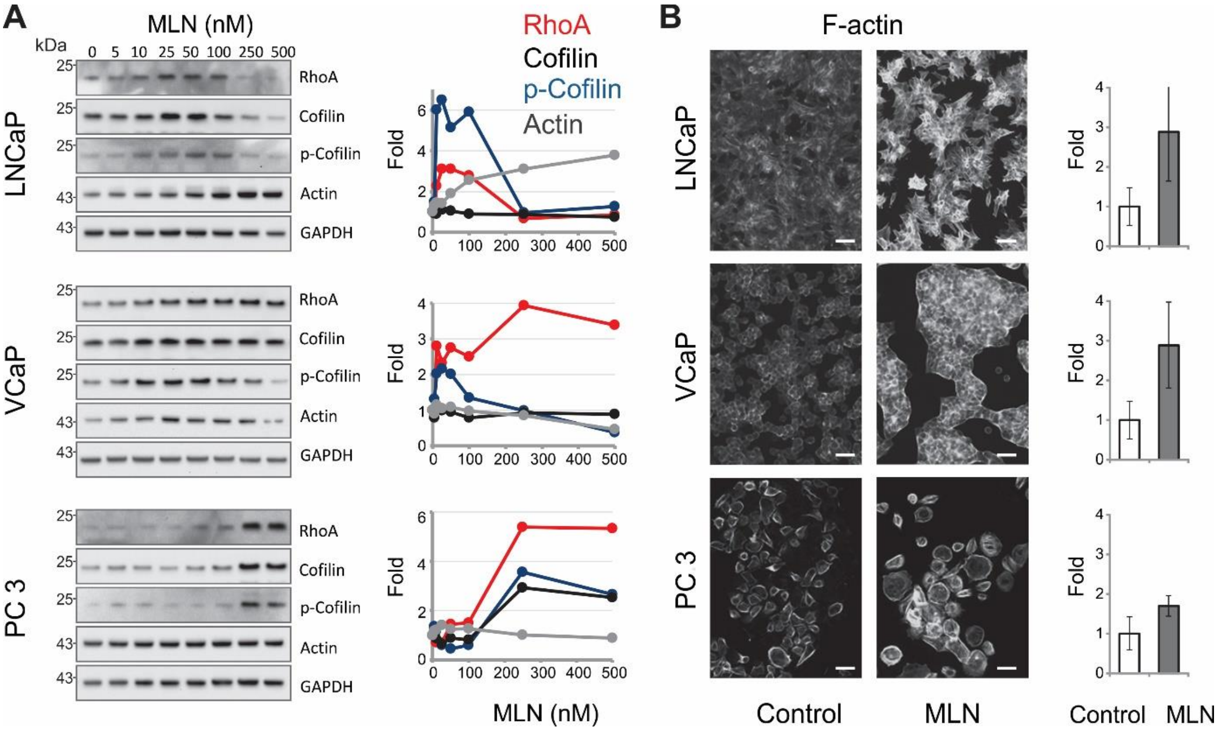

Supplementary Figure S3. MLN upregulates RhoA and stabilizes F-actin. (A) Dose-dependent effect of MLN on RhoA-Cofilin-Actin levels shown by western blots with the corresponding quantifications on the right. (B) Actin polymerization induced by 100 nM MLN in PCa cells with the corresponding quantifications on the right. DMSO was used as a vehicle control. Scale bar = 50 µm.

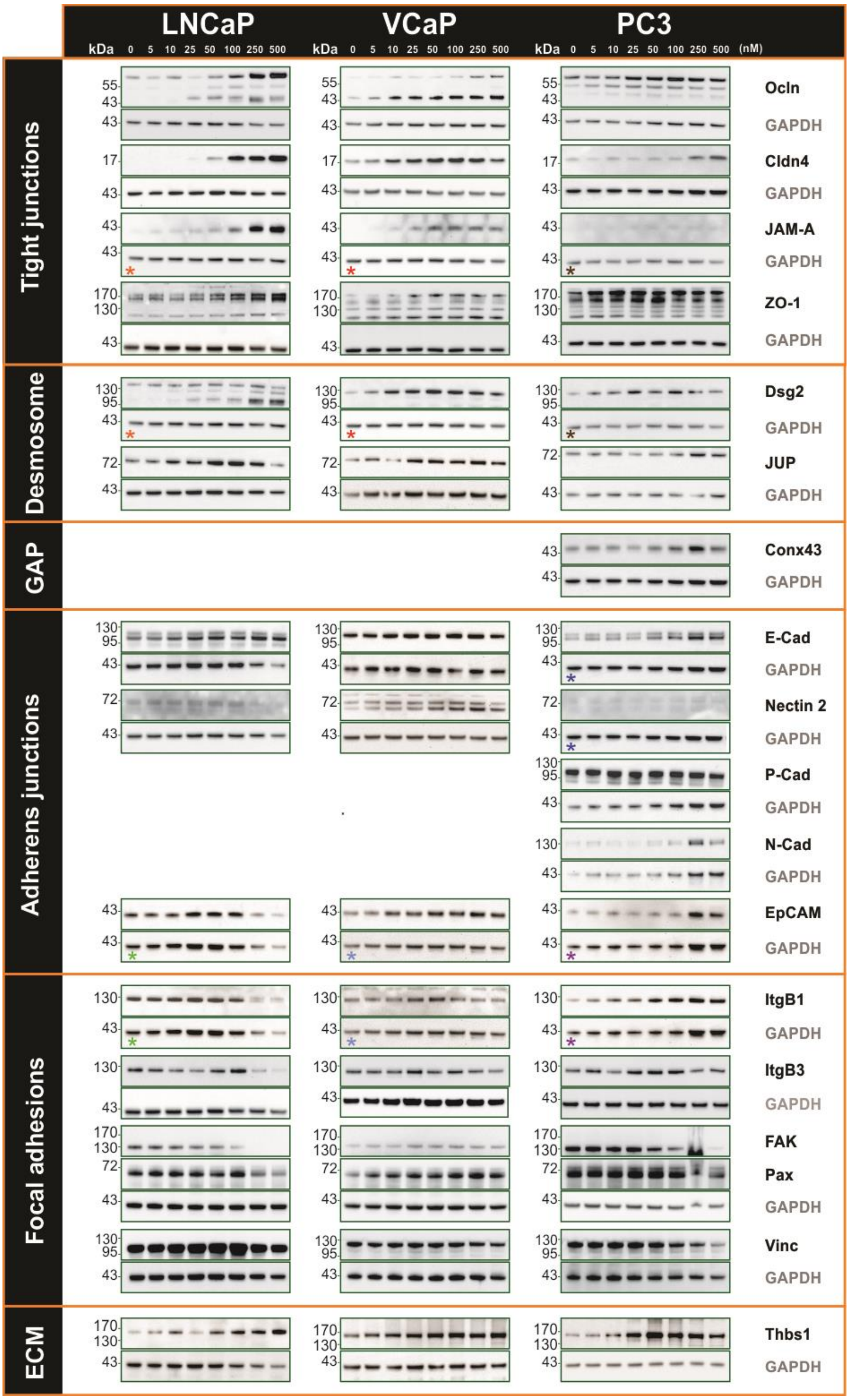

Supplementary Figure S4. Dose-dependent effect of MLN on protein expression. MLN concentration is indicated on the top. The asterisks indicate the membranes used for the analysis of two different proteins with the same GAPDH as a control.

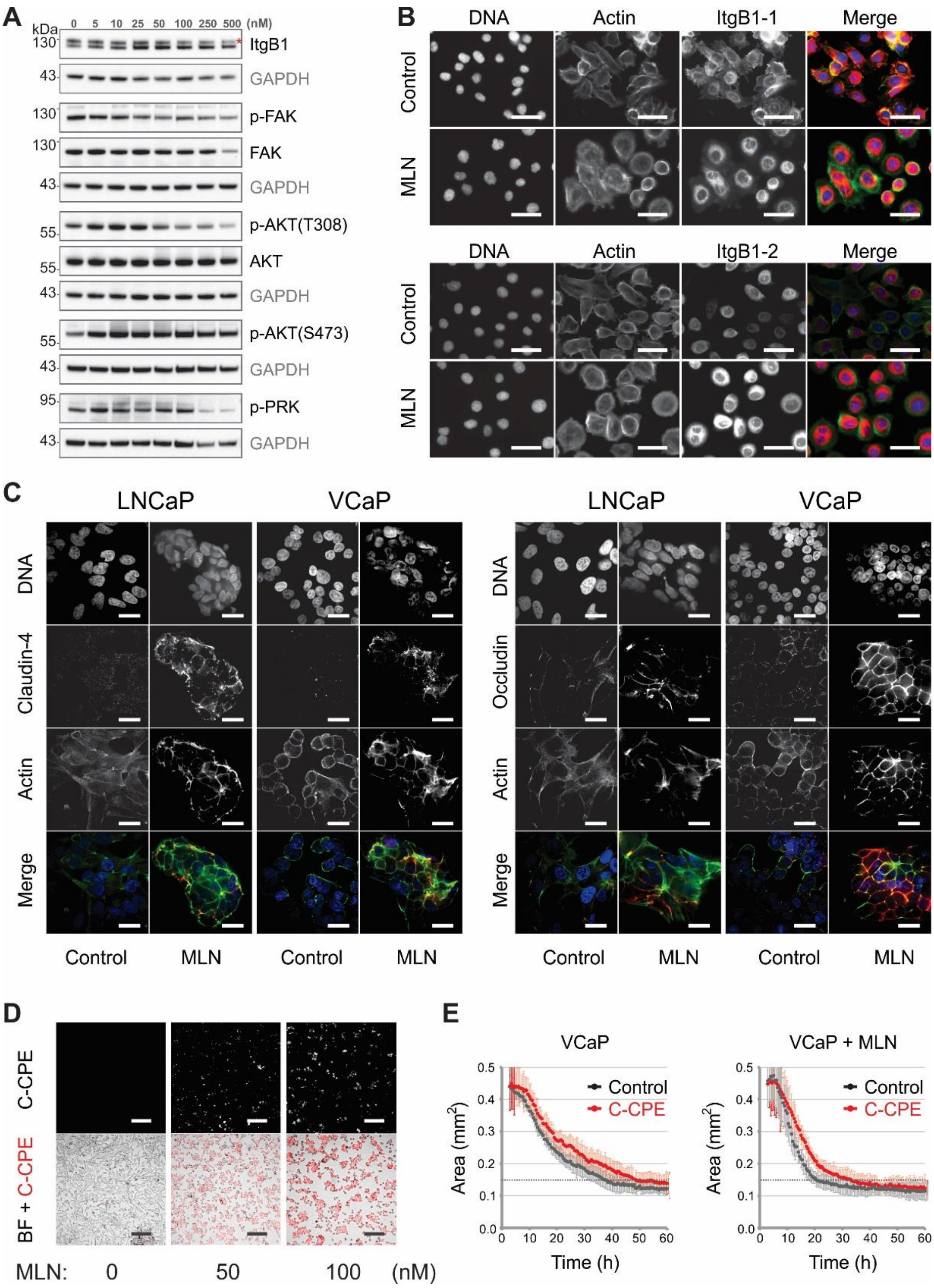

Supplementary Figure S5. MLN affects distinct membrane proteins in PCa cells. (A) Dose-dependent effect of MLN on ItgB1 expression and protein kinase signaling in PC3 cells. To avoid the eventual loss of the mature ItgB1 form (red asterisk), the cells were quenched by Laemmli buffer directly in the plates. (B) Expression of ItgB1 in PC3 cells treated with 100 nM MLN or DMSO (control) analyzed by two different ItgB1 antibodies (MAR4 and 9EG7). Scale bar = 50 µm. (C) Cell morphology and expression of Cldn4 and Ocln proteins in LNCaP and VCaP cells treated with 100 nM MLN. Scale bar = 25 µm. (D) Binding of Cy3-labeled C-CPE to control and MLN-treated LNCaP cells analyzed by microscopy. Fluorescence and combined with bright field (BF) images are shown. Scale bar = 200 µm. (E) Effect of 200 µg/ml C-CPE on VCaP spheroid assembly with or without 100 nM MLN (mean ± S.D., n=6). In all experiments with MLN, DMSO was used as a vehicle control.

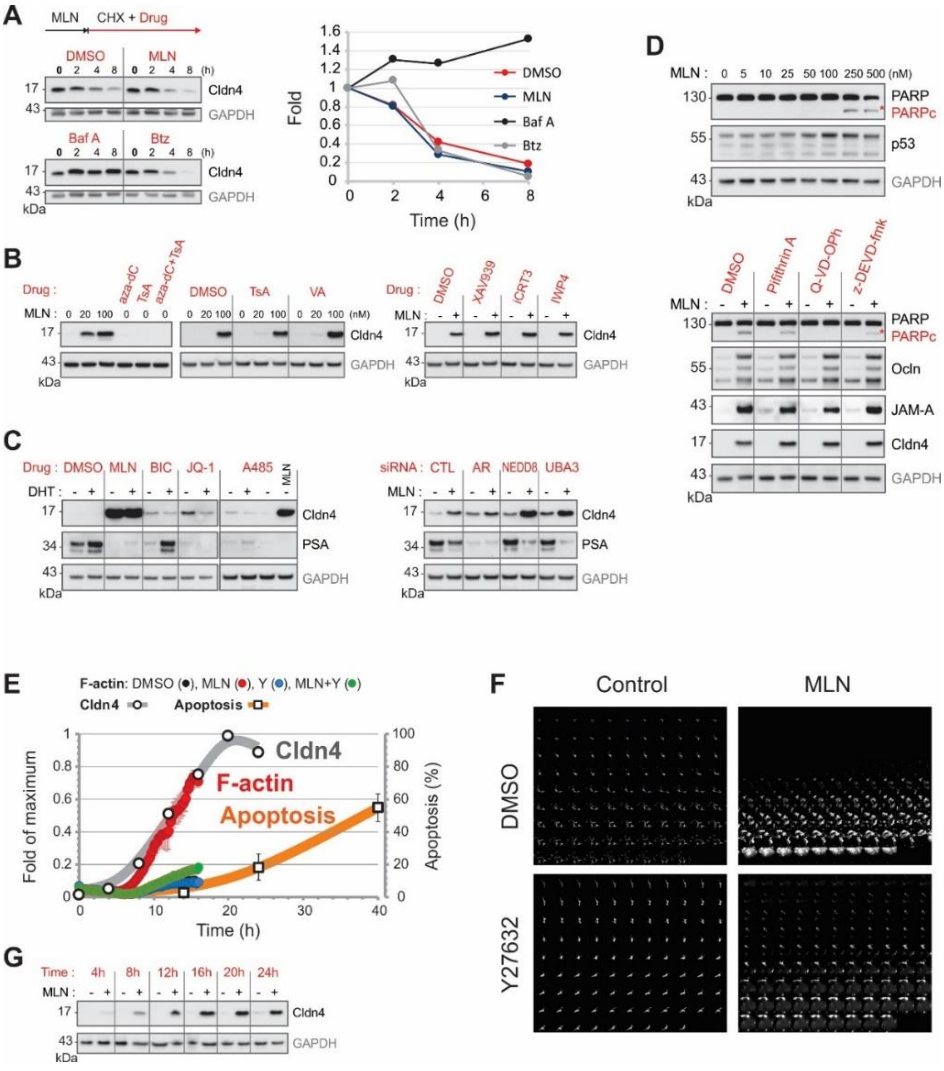

Supplementary Figure S6. The pathways involved in upregulation of TJ expression by MLN. (A) Cycloheximide (CHX) chase analysis of Cldn4 degradation in LNCaP cells. Conditions: 100 nM MLN, 5 nM bafilomycin A (Baf A, autophagy inhibitor), and 100 nM bortezomib (Btz, proteasome inhibitor). The quantification of the blots is shown on the right. Similar results were obtained for occludin except that Btz had a partial stabilizing effect (not shown) (B) Effect of the chromatin remodeling drugs and the inhibitors of Wnt/β-Cat pathway on the stimulation of Cldn4 expression by MLN. Conditions: 10 µM 5-Aza-2'-deoxycytidine (aza-dC), 500 nM Trichostatin A (TsA), 2 mM Valproic acid (VA), 5 µM XAV939, 5µM iCRT3, and 2µM IWP4. (C) Effect of AR signaling on Cldn4 expression in LNCaP cells. Conditions: 10 nM DHT, 100 nM MLN, 10 µM bicalutamide (BIC), 100 nM JQ-1, and 1 µM A485. Conclusions: AR inhibition increases the level of Cldn4 (but to a much lesser extent than MLN) that is counteracted by DHT. MLN inhibits AR in LNCaP cells (seen by PSA expression). The inhibition of AR by A485 has no effect on the stimulation of Cldn4 expression by MLN. Panels on the right show that AR knockdown does not affect Cldn4 expression, whereas the inhibition of neddylation by depleting NEDD8 or UBA3 stimulates it. Of note, in contrast to MLN, NEDD8 and UBA3 depletions alone are not sufficient to block AR signaling (seen by PSA expression). (D) Dose-dependent apoptosis activation by MLN revealed by p53 induction and PARP cleavage (red asterisk). The bottom panels show that complete (20 µM Q-VD-OPh) or partial (20 µM z-DEVD-fmk) inhibition of the apoptotic program (seen by PARP cleavage) do not affect the stimulation of TJ expression by 100 nM MLN. (E) Time course of actin polymerization, Cldn4 expression and apoptosis after the addition of 100 nM MLN to LNCaP cells. Actin polymerization was followed by time-lapse confocal microscopy using CellLight™ Actin-RFP reagent (see Supplementary Figure S6F for representative images) and quantified using Fiji (mean ± S.D., n=5). Of note, 10 µM Y27632 significantly suppresses the stimulation of actin polymerization induced by MLN. Cldn4 expression was measured by western blot (see Supplementary Figure S6G). Apoptosis was analyzed using the CellEvent reagent.

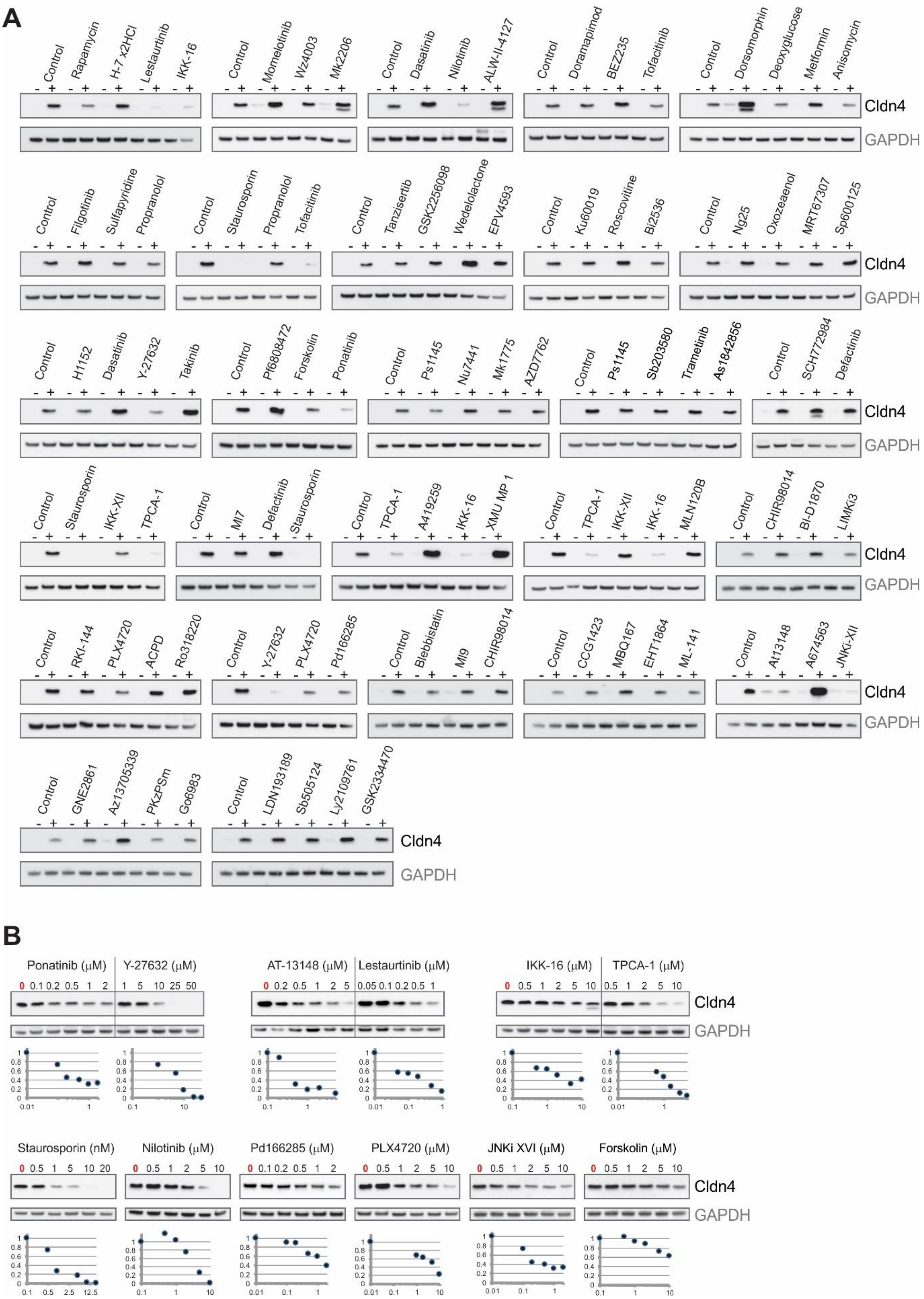

Supplementary Figure S7. Effect of drugs on Cldn4 expression. (A) Representative western blots show the effect of different drugs on Cldn4 expression with or without 100 nM MLN (see Supplementary Table S1 for further information). DMSO was used as a vehicle control. (B) Dose-response curves for selected TJSR inhibitors.

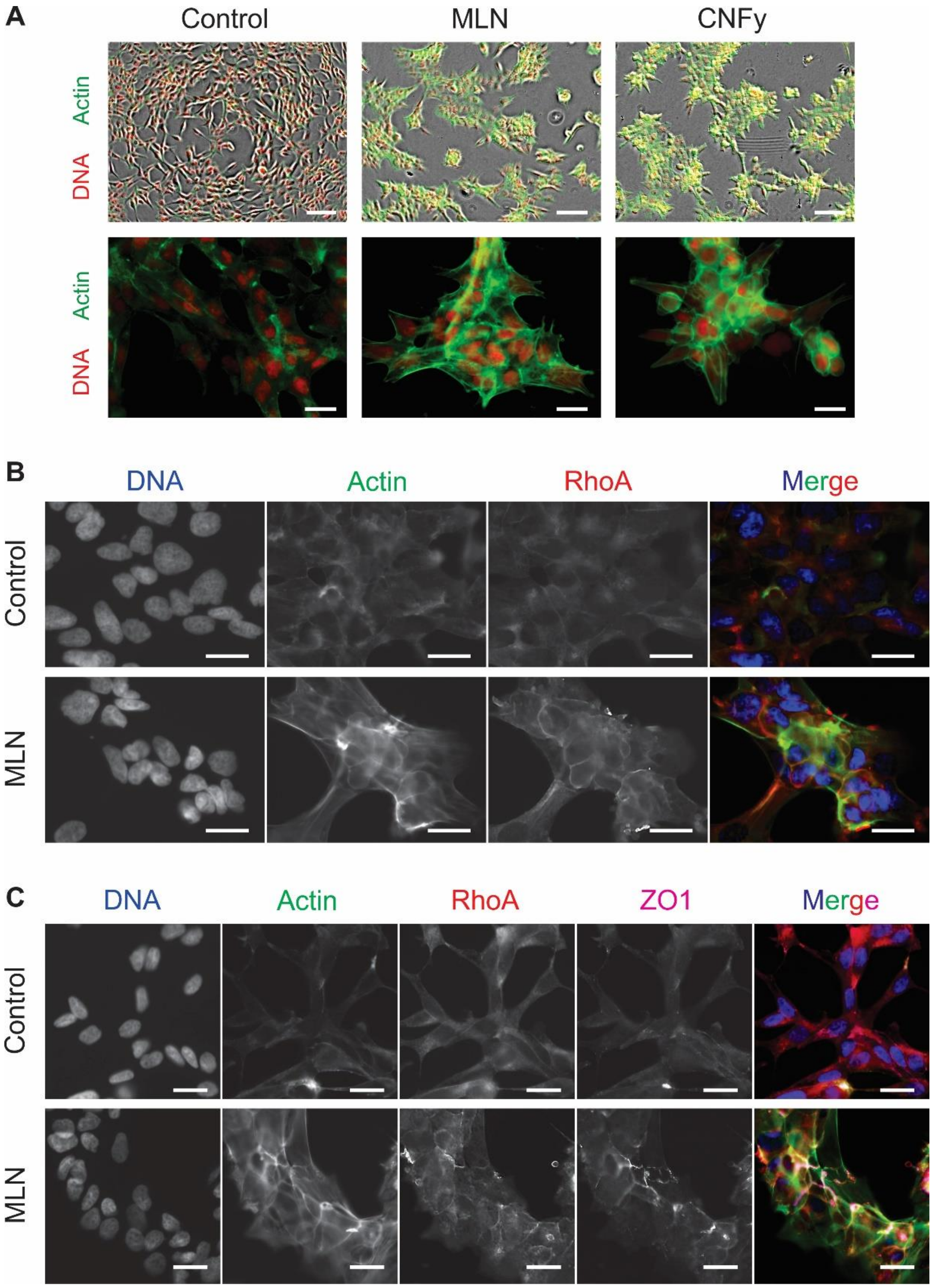

Supplementary Figure S8. Effect of MLN on LNCaP cell morphology and protein localization. (A) Comparison of the effect of 100 nM MLN and 1 nM CNFy on LNCaP cell morphology. Scale bars = 100 µm (top) and 25 µm (bottom). (B) Localization of RhoA in control (DMSO) and 100 nM MLN treated LNCaP cells. Scale bar = 25 µm. (C) Localization of ZO1 and RhoA in control (DMSO) and 100 nM MLN treated LNCaP cells. Scale bar = 25 µm.

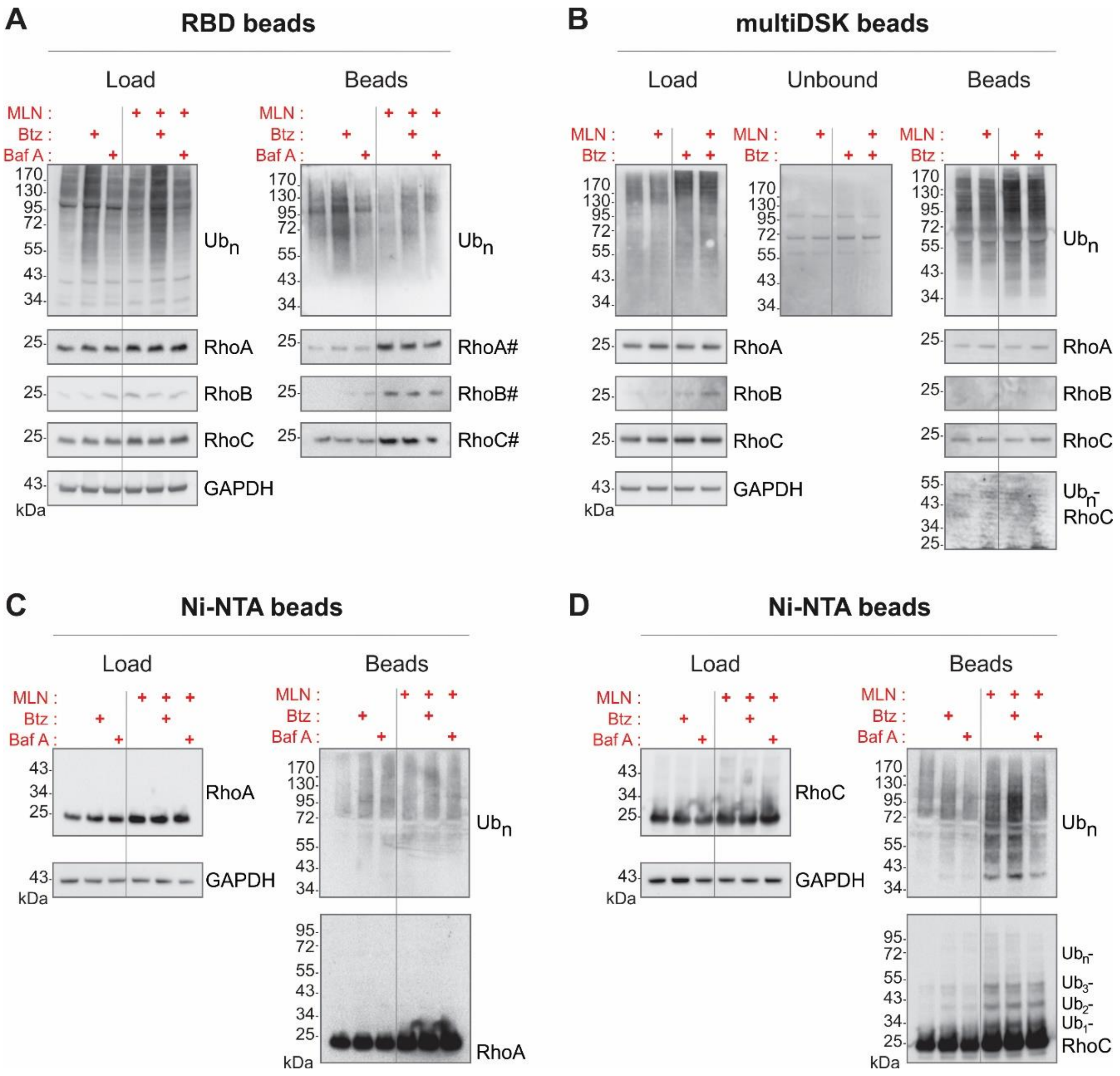

Supplementary Figure S9. Effect of proteolysis inhibitors on Rho stability and ubiquitylation in LNCaP cells. (A) Analysis of active Rho-GTPs using Rhotekin Rho Binding Domain (RBD) pull-down assay. The cells were treated with 50 nM MLN or DMSO as a vehicle control for 20 h and then with 500 nM bortezomib (Btz) or 5 nM bafilomycin A (Baf A) for 4 h, where indicated. The active GTP-bound Rho isoforms (hash-tagged) were isolated on RBD beads and analyzed by western blotting using isoform specific antibodies. In parallel, the level of ubiquitin conjugates (Ubn) was assessed using P4D1 anti-ubiquitin antibody. Although some amounts of Ubn were isolated on the beads, the method did not enable the detection of endogenous levels of ubiquitylated Rho-GTPs using Rho-specific antibodies. (B) Analysis of ubiquitylated proteins by capturing on a ubiquitin-specific affinity resin multiDSK. The cells were treated with drugs as described above and ubiquitin conjugates and bound proteins were isolated on multiDSK beads. The capture of ubiquitylated proteins was almost 100% effective as judged by western blotting of the unbound protein fraction (in the middle). Small amounts of RhoA and RhoC were isolated on the beads but endogenous ubiquitin conjugates were detected only for RhoC (by extending the exposure time). (C,D) Analysis of Rho modifications using metal-affinity isolation of his6-tagged Rho proteins in denaturing conditions. The cells were transfected for 24 h with pcDNA3.1 plasmids coding for his6-RhoA (Figure S9C) or his6-RhoC (Figure S9D) and then were treated with drugs as described above. The cells were lysed in denaturing buffer and his6-tagged Rho proteins were isolated on Ni-NTA resin and analyzed by western blotting using specific antibodies. All drugs upregulated the ubiquitin conjugates, particularly MLN, which induced high levels of ubiquitylated Rho species detectible by Rho-specific antibodies. The effect was much more pronounced with his6-RhoC (Figure S9D).

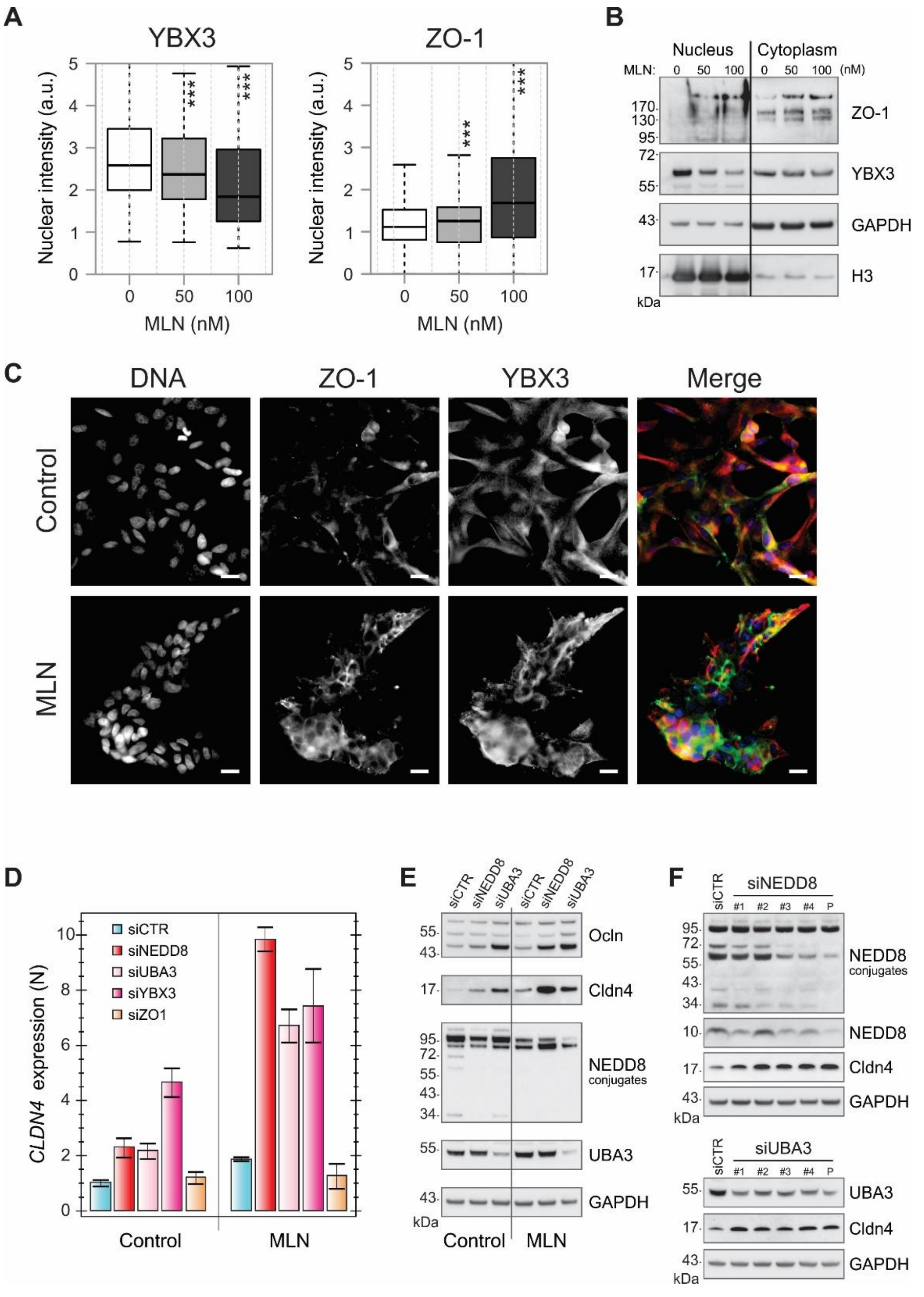

Supplementary Figure S10. Characterization of TJSR pathway. (A) Nuclear localization of ZO1 and YBX3 proteins in LNCaP cells measured by automated fluorescence microscopy. Statistical significance: ***-p < 0.001. (B) Cell fractionation analysis of the localization of ZO1 and YBX3 proteins in control and MLN treated LNCaP cells. DMSO was used as a vehicle control. (C) Immunofluorescence images show the localization of ZO1 and YBX3 proteins in control (DMSO) and 100 nM MLN treated LNCaP cells. Scale bar = 25 µm. (D) Effect of neddylation inhibition and TJSR pathway modification on *CLDN4* transcription. The indicated genes were knocked down in LNCaP cells using SmartPool siRNAs. After 24 h, the cells were treated with DMSO (control) or 20 nM MLN for additional 24 h and analyzed by RT–qPCR. (E) Effect of neddylation inhibition on TJ protein expression. The cells were treated as described in Figure S10D and analyzed by western blotting. (F) Effect of the individual and SmartPool (P) siRNAs on the target proteins and Cldn4 expression.

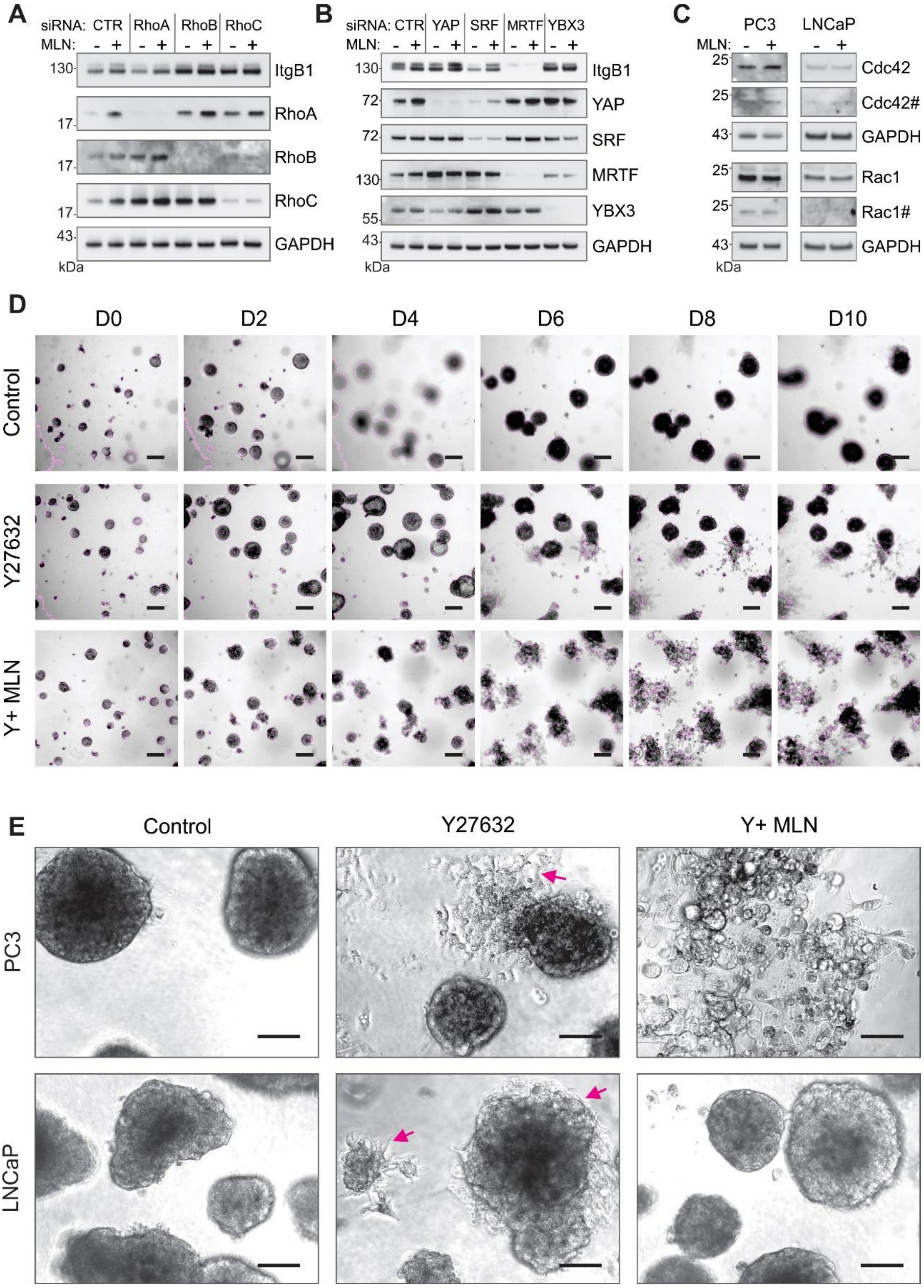

Supplementary Figure S11. Role of different factors in MLN induced phenotypes. (A,B) Effect of knockdowns of Rho isoforms and specific mechano-responsive transcriptional regulators on the upregulation of ItgB1 expression by 100 nM MLN in PC3 cells. (C) Effect of 100 nM MLN on Rac1 and Cdc42 GTPases in PC3 and LNCaP cells. The active GTP-bound isoforms are hash-tagged. (D) Effect of 100 nM MLN and 10 µM Y27632 (Y) on PC3 tumoroid growth over 10 days. DMSO was used as a vehicle control. Scale bar = 200 µm. (E) Representative images demonstrate mesenchymal invasion induced by 10 µM Y27632 in PCa tumoroids (magenta arrows) and distinct effects of 100 nM MLN on tumoroid morphology. Scale bar = 100 µm.

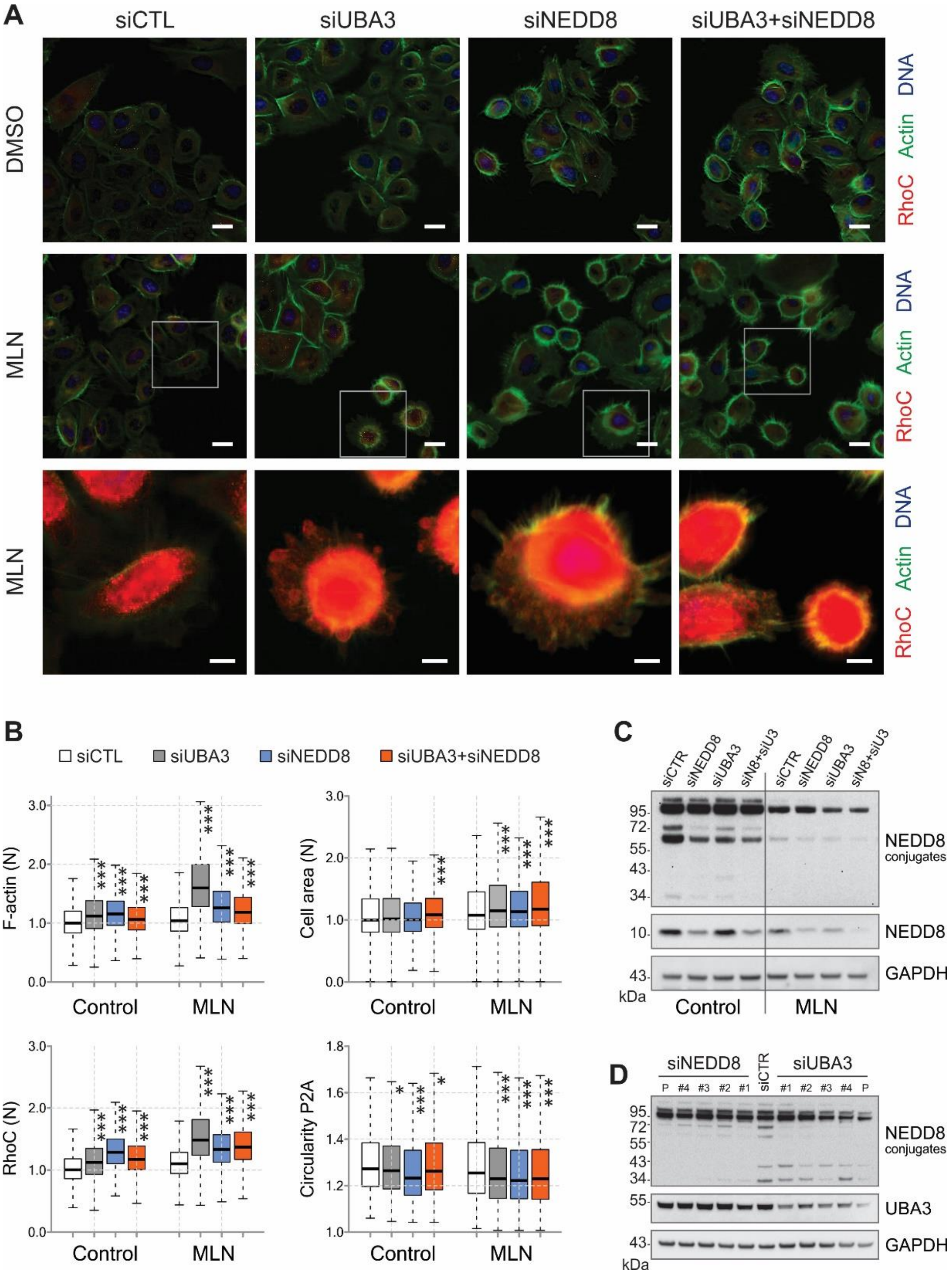

Supplementary Figure S12. Effect of neddylation inhibition on PC3 cell morphology. (A) UBA3 and NEDD8 depletion significantly affects the morphology of PC3 cells, particularly when combined with a suboptimal dose of MLN (20 nM). The indicated genes were knocked down using SmartPool siRNAs. After 24 h, the cells were treated with DMSO (control) or 20 nM MLN for additional 24 h and analyzed by immunofluorescence microscopy using CellInsight automated platform. Scale bar = 25 µm and 10 µm (zooms). (B) Quantitative analysis of the effect of the gene knockdowns shown in Figure S12A. Average cell intensity of F-actin and RhoC as well as cell area and circularity (P2A) were measured using HCS Studio software and analyzed by "R". Statistical significance: *-p < 0.05 and ***-p < 0.001. (C) Western blot analysis of neddylation inhibition by SmartPool siRNAs. The conditions were as in Figure S12A. (D) Effect of the individual and SmartPool (P) siRNAs on NEDD8 and UBA3 protein expression.

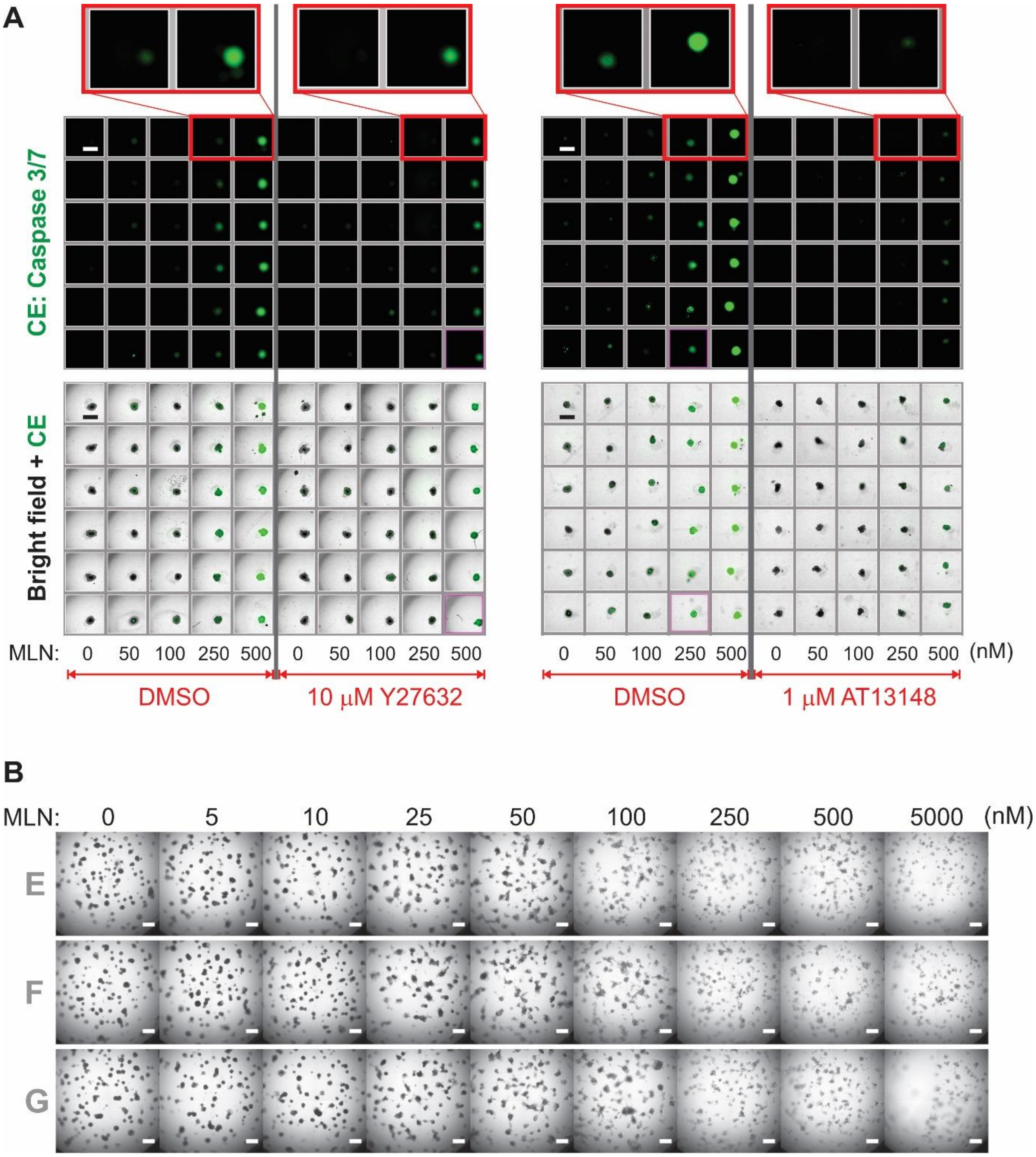

Supplementary Figure S13. Role of Rho signaling in MLN induced apoptosis and invasion. (A) Effect of ROCK inhibitors on MLN induced apoptosis in small (<250 µm) LNCaP spheroids measured by CE fluorescence. Scale bar = 500 µm. (B) Representative experiment (in triplicate) used for the analysis of dose-dependent invasion in PC3 tumoroids (day 4). Scale bar = 500 µm.

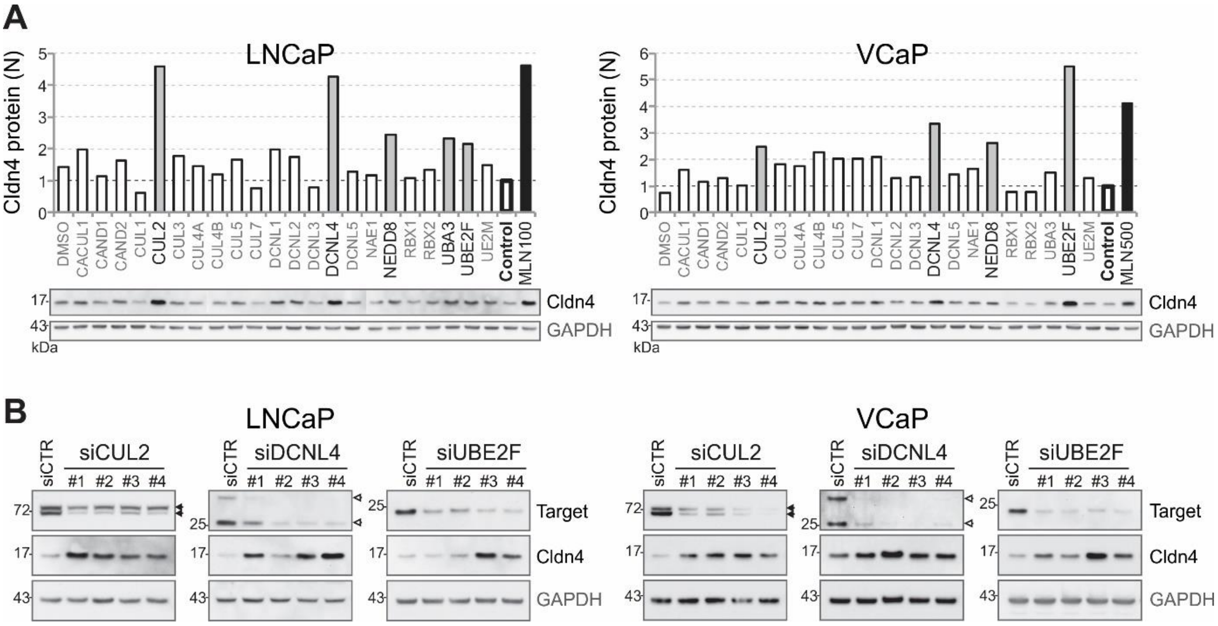

Supplementary Figure S14. TJSR regulators are potential therapeutic targets. (A) Functional genomic siRNA screen for TJSR regulators in LNCaP and VCaP cells. The indicated genes were knocked down using SmartPool siRNAs, and the level of Cldn4 protein was measured by western blot. (B) Hit validation using four individual siRNAs. Western blots show the extinction of the target proteins and the corresponding Cldn4 level. Double black arrowheads indicate native and neddylated CUL2 protein. Double white arrowheads indicate DCNL4 isoforms.

Supplementary Tables

Supplementary Table S1. List of the drugs.

#	Drug	Main target	IC50 reported	Concentration	Cldn4 level	IC50-Cldn4	CAS number	Drug source	Reference
1	**Control-MLN4924**	NAE1	5 nM	100 nM	1,00		905579-51-3	MedChemExpress	HY-70062
2	**XMU MP 1**	MST1/2	71/ 38 nM	5 μM	4,20		2061980-01-4	SIGMA	SML2233
3	**Wedelolactone**	NFkB (IKKα/γ) / caspase-11		25 μM	3,97		524-12-9	SIGMA	W4016
4	**EVP4593**	NFkB/ TNFα	11/ 7 nM	5 μM	3,96		545380-34-5	SIGMA	SML0579
5	**Dorsomorphin**	AMPK/ BMP pathway (ALK2/3/6)	101 nM (AMPK)	5 μM	3,48		866405-64-3	SIGMA	P5499
6	**AZ13705339**	PAK1/2	0.3/ 6 nM	5 μM	3,44		2016806-57-6	Tocris Bioscience	6177/10
7	**Takinib**	TAK1	9 nM	5 μM	2,67		1111556-37-6	SIGMA	SML2216
8	**ALW-II-4127**	Eph2	11 nM	5 μM	2,55		1186206-79-0	MedChemExpress	HY-18007
9	**MK-2206**	AKT1/2/3	8/ 12/ 65 nM	5 μM	2,20		1032350-13-2	Santa Cruz Biotechnology	sc-364537
10	**A-419259**	Hck and other Src family kinases (SFKs)	0.4 nM	5 μM	2,14		364042-47-7	SIGMA	SML0446
11	**Ro 31-8220**	PKCα/βI/βII/γ/ε	5/ 24/ 14/ 27/ 24 nM	1 μM	2,10		138489-18-6	Santa Cruz Biotechnology	sc-200619
12	**SCH 772984**	ERK1/2	4/ 1 nM	5 μM	2,08		942183-80-4	ApexBio Technology	AOB6425
13	**BI-D1870**	RSK1/2/3/4	3/ 24/ 18/ 15 nM	5 μM	1,98		501437-28-1	Santa Cruz Biotechnology	sc-397022
14	**Dasatinib**	ABL/ SRC/ LCK/ YES/ KIT etc	1/ 0.5/ 0.4/ 0.5/ 5 nM	1 μM	1,92		302962-49-8	Santa Cruz Biotechnology	sc-358114
15	**GNE2861**	PAK4/5/6	7.5/ 36/ 126 nM	5 μM	1,90		1394121-05-1	MedChemExpress	HY-12632
16	**MBQ167**	Rac/ Cdc42	103/ 78 nM	5 μM	1,88		2097938-73-1	MedChemExpress	HY-112842
17	**SP600125**	JNK1/2/3	40/ 40/ 90 nM	5 μM	1,88		129-56-6	Santa Cruz Biotechnology	sc-200635
18	**Go6983**	PKCα/β/γ/δ	7/ 7/ 6/10 nM	5 μM	1,78		133053-19-7	MedChemExpress	HY-13689
19	**Momelotinib**	JAK1/2	11/18 nM	5 μM	1,77		1056634-68-4	Santa Cruz Biotechnology	sc-364733
20	**Defactinib**	FAK/ PYK2	0.6/0.6 nM	5 μM	1,65		1345713-71-4	AdooQ® Bioscience	A11929-2
21	**A-674563**	AKT1/ PKA /CDK2	11/ 16/ 46 nM	5 μM	1,63		552325-73-2	VWR/Cayman Chemical	CAYM18715
22	**PF6808472**	multiple kinases		1 μM	1,58		2088112-70-1	SIGMA	PZ0306
23	**Roscovitine**	Cdc2/ CDK2/5	0.6/ 0.7/ 0.2 μM	5 μM	1,57		186692-46-6	SIGMA	R7772
24	**LY2109761**	TGF-β RI/II	38/ 300 nM	5 μM	1,53		700874-71-1	Santa Cruz Biotechnology	sc-396262
25	**BEZ235**	PI3K p110α/γ/δ/β/ mTOR p70S6K/ ATR)	4/ 5/ 7 / 75/ 6/ 21 nM	5 μM	1,50		915019-65-7	Santa Cruz Biotechnology	sc-364429
26	**GSK2256098**	FAK	0.4 nM	5 μM	1,49		1224887-10-8	VWR/Cayman Chemical	CAYM22955-1
27	**MRT67307**	IKKε/ TBK1	160/ 19 nM	5 μM	1,45		1190378-57-4	SIGMA	SML0702
28	**Metformin**	AMPK activator		5 mM	1,41		1115-70-4	SIGMA	PHR1084
29	**KU-60019**	ATM	6.3 nM	5 μM	1,40		925701-49-1	SIGMA	SML1416
30	**ACPD**	aPKC		5 μM	1,36		4056-73-9	SIGMA	156493
31	**Filgotinib**	JAK1/2/3/ TYK2	10/ 28/ 810/ 116 nM	5 μM	1,34		1206161-97-8	MedChemExpress	HY-18300
32	**EHT1864**	Rac1/1b/2/3	40/ 50/ 60/ 250 nM	5 μM	1,29		754240-09-0	MedChemExpress	HY-16659
33	**GSK2334470**	PDK1	10 nM	5 μM	1,27		1227911-45-6	SIGMA	SML0217
34	**BI2536**	Plk1	0.8 nM	10 nM	1,25		755038-02-9	ApexBio Technology	A3965
35	**LDN193189**	ALK1/2/3/6	0.8/ 0.8/ 5.3/ 16.7 nM	5 μM	1,24		1435934-00-1	VWR/Cayman Chemical	CAYM19396-1
36	**NG25**	TAK1/ MAP4K2	149/ 21.7 nM	5 μM	1,22		1315355-93-1	SIGMA	SML1332
37	**AZD7762**	Chk1/2	5/ 5 nM	5 μM	1,18		1246094-78-9	AdooQ® Bioscience	A10113-5
38	**NU7441**	DNA-PK	14 nM	5 μM	1,18		503468-95-9	ApexBio Technology	A8315
39	**Tanzisertib**	JNK1/2/3	61/ 7/ 6 nM	5 μM	1,16		899805-25-5	MedChemExpress	HY-15495
40	**SB505124**	ALK4/5	129/ 47 nM	5 μM	1,16		694433-59-5	MedChemExpress	HY-13521-S
41	**CCG1423**	SRF-mediated transcription		5 μM	1,12		285986-88-1	SIGMA	SML0987
42	**WZ4003**	NUAK1/2	20/ 100 nM	5 μM	1,11		1214265-58-3	Tocris Bioscience	5177
43	**PKzPSm**	aPKC isozyme PKMζ pseudo-substrate	1 μM	10 μM	1,08		863987-12-6	Santa Cruz Biotechnology	sc-397537
44	**ML141**	Cdc42	200 nM	5 μM	1,07		71203-35-5	MedChemExpress	HY-12755
45	**H-7 x2HCl**	PKC/ PKG/ PKA		5 μM	1,05		84477-87-2	ApexBio Technology	BS0093
46	**Doramapimod**	p38α/β/γ/δ	38/ 65/ 200/ 520 nM	5 μM	1,03		285983-48-4	Santa Cruz Biotechnology	sc-300502
47	**(5Z)-7-Oxozeaenol**	TAK1/ VEGF-R2/ ERK2	8/ 52/ 80 nM	1 μM	1,00		253863-19-3	Merck	499610
48	**ML-7**	MLCK	0.3 μM	5 μM	0,95		110448-33-4	SIGMA	i2764
49	**Sulfapyridine**	NIK/ IKKα		40 μM	0,91		144-83-2	SIGMA	S6252
50	**CHIR98014**	GSK-3α/β	0.6/0.6 nM	1 μM	0,90		252935-94-7	ApexBio Technology	AOB6865
51	**MK1775**	Wee1	5 nM	0.1 μM	0,89		955365-80-7	AdooQ® Bioscience	A10599
52	**H1152**	ROCK1/2	1.6/ 12 nM	1 μM	0,86		872543-07-6	Merck	555550
53	**Trametinib**	MEK1/2	0.9/ 1.8 nM	5 μM	0,84		871700-17-3	Santa Cruz Biotechnology	sc-364639
54	**ML120B**	IKKβ	60 nM	5 μM	0,84		783348-36-7	MedChemExpress	HY-15473
55	**LIMKi3**	LIMK1/2	7/ 8 nM	5 μM	0,82		1338247-35-0	SIGMA	435930
56	**SB203580**	p38/ AKT	0.3/ 0.5 μM	5 μM	0,81		152121-47-6	Santa Cruz Biotechnology	sc-3533
57	**IKK-XII**	IKKα/β	500/ 100 nM	5 μM	0,81		928655-63-4	Merck	401491
58	**RKI-1447**	ROCK1/2	14.5/ 6 nM	5 μM	0,79		1342278-01-6	MedChemExpress	HY-15755-S
59	**Propranolol**	NIK/ IKKα		60 μM	0,78		13071-11-9	SIGMA	P0689
60	**PLX4720**	B-Raf(V600E)	13 nM	5 μM	0,77	5 μM	918505-84-7	ApexBio Technology	AOB87700-5
61	**Tofacitinib**	JAK1/2/3	112/ 20/ 1 nM	5 μM	0,74		540737-29-9	SIGMA	PZ0017
62	**ML-9**	MLCK	3 μM	5 μM	0,67		105637-50-1	SIGMA	C1172
63	**PD 166285**	c-Src/ FGFR1)/ PDGFRβ and other RTK	8/ 39/ 98 nM	0.5 μM	0,63	0.5 μM	212391-63-4	SIGMA	PZ0116
64	**PS1145**	IKK	88 nM	5 μM	0,61		431898-65-6	Santa Cruz Biotechnology	sc-301621
65	**2-Deoxy-D-glucose**	AMPK activator		1 mM	0,61		154-17-6	SIGMA	D8375
66	**AS1842856**	Foxo1	33 nM	5 μM	0,55		836620-48-5	MedChemExpress	HY-100596-s
67	**Y-27632**	ROCK1/2	220/ 300 nM	5 μM	0,54	5 μM	129830-38-2	SIGMA	Y0503
68	**Blebbistatin**	Myosin II	2 μM	10 μM	0,52		856925-71-8	SIGMA	B0560
69	**Anisomycin**	p38 activator		0.1 μM	0,47		22862-76-6	SIGMA	A9789
70	**Forskolin**	Adenylate cyclase activator		10 μM	0,44	10 μM	66575-29-9	SIGMA	93049
71	**Rapamycin**	mTOR	0.1 nM	20 nM	0,40		53123-88-9	SIGMA	R8781
72	**AT-13148**	ROCK1/2/ PKA/p70S6K and other AGC kinases	6/ 4/ 3/ 8 nM	5 μM	0,25	0.3 μM	1056901-62-2	VWR/Cayman Chemical	CAYM21597-1
73	**IKK-16**	IKKα/β	200/ 40 nM	5 μM	0,24	2 μM	1186195-62-9	AdooQ® Bioscience	A12836-10
74	**TPCA-1**	IKKα/β	400/ 18 nM	5 μM	0,19	1 μM	507475-17-4	MedChemExpress	HY-10074
75	**Nilotinib**	ABL/ KIT/ PDGFRα etc	20/ 100/ 0.5 nM	5 μM	0,17	3 μM	641571-10-0	Acros Organics	15851032
76	**Ponatinib**	ABL/ PDGFRα/ VEGFR2/ FGFR1/ Src	0.4/ 1/ 1.5/ 2/ 5 nM	1 μM	0,13	0.2 μM	943319-70-8	Santa Cruz Biotechnology	sc-396905
77	**JNKi XVI**	JNK1/2/3	5/ 19/ 1 nM	5 μM	0,12	0.3 μM	1410880-22-6	Cayman Chemical	CAYM18096-1
78	**Lestaurtinib**	JAK2/ FLT3/ TrkA/ AurA/B	1/ 3/ 25/ 8/ 2 nM	0.5 μM	0,04	0.2 μM	111358-88-4	VWR/Cayman Chemical	CAYM12094-500
79	**Staurosporine**	pan-kinase		50 nM	0,01	2 nM	62996-74-1	ApexBio Technology	AOB1831
80	**Bafilomycin A1**	Vacuolar H+ ATPase (V-ATPase)	0.44 nM	5 nM			88899-55-2	AdipoGen	BVT-0252-M001
81	**Bortezomib**	Proteasome	0.6 nM	100 nM			179324-69-7	MedChemExpress	HY-10227
82	**5-Aza-2′-Deoxycytidine**	DNMT1		10 μM			2353-33-5	Santa Cruz Biotechnology	sc-202424
83	**Trichostatin A**	HDAC class I and II	3.4 nM	500 nM			58880-19-6	SIGMA	T8552
84	**Valproic acid**	HDAC		2 mM			99-66-1	SIGMA	PHR1061
85	**XAV939**	TNKS1/2	11/ 4 nM	5 μM			284028-89-3	SIGMA	X3004
86	**iCRT3**	Wnt/β-catenin signaling	8 nM	5 μM			901751-47-1	SIGMA	SML0211
87	**IWP4**	Wnt/β-catenin signaling	25 nM	2 μM			686772-17-8	SIGMA	SML1114
88	**Bicalutamide**	androgen receptor (AR)	160 nM	10 μM			90357-06-5	Santa Cruz Biotechnology	sc-202976
89	**JQ-1**	BRD2/3/4 bromodomains	20-200 nM	100 nM			1268524-70-4	ApexBio Technology	A1910
90	**A-485**	p300/CBP HAT	10/ 3 nM	1 μM			1889279-16-6	Tocris Bioscience	6387/5
91	**Pifithrin-alpha**	p53 activator		5 μM			63208-82-2	AdooQ® Bioscience	A12451-5
92	**Q-VD-OPh hydrate**	Caspases 1/ 3/ 8/ 9	25-400 nM	20 μM			1135695-98-5	ApexBio Technology	A1901-1
93	**Z-DEVD-FMK**	Caspases 3/ 6/ 7/ 8/ 10	18 μM	20 μM			210344-95-9	MedChemExpress	HY-12466

Supplementary Table S2. List of the antibodies.						
#	Protein	Source	Reference	Application	Host	Type
1	Actin	Merck Millipore	MAB1501	WB	mouse	m
2	AKT	Cell Signaling	4691P	WB	rabbit	m
3	AKT (phospho-S473)	Cell Signaling	4058T	WB	rabbit	m
4	AKT (phospho-T308)	Cell Signaling	13038P	WB	rabbit	m
5	Cdc42	Santa Cruz	sc-8401	WB	mouse	m
6	Claudin 4	Santa Cruz	sc-376643	WB, IF	mouse	m
7	Cofilin	Abcam	ab42475	WB	rabbit	p
8	Cofilin (phospho-S3)	SIGMA	SAB4504370	WB	mouse	m
9	Connexin 43	Santa Cruz	sc-271837	WB	mouse	m
10	Cullin 2	Invitrogen	511800	WB	rabbit	p
11	DCNL4	MRC (Dundee)	S997C	WB	sheep	p
12	Desmoglein 2	Santa Cruz	sc-80663	WB	mouse	m
13	E-Cadherin	BD Biosciences	610181	WB	mouse	m
14	EpCAM	Santa Cruz	sc-25308	WB	mouse	m
15	FAK	BD Biosciences	610087	WB	mouse	m
16	FAK (phospho-Y397)	Cell Signaling	8556T	WB	rabbit	m
17	GAPDH	Santa Cruz	sc-47724	WB	mouse	m
18	Histone H3	Invitrogen	701517	WB	rabbit	m
19	Integrin β1	BD Biosciences	550531 (9EG7)	WB, IF	mouse	m
20	Integrin β1 (Hu-CD29-PE)	BD Biosciences	561795 (MAR4)	IF	mouse	m
21	Integrin β3	Santa Cruz	sc-46655	WB	mouse	m
22	JAMA	Santa Cruz	sc-53623	WB	mouse	m
23	JUP (Plakoglobin)	Santa Cruz	sc-8415	WB	mouse	m
24	MRTF/MKL1	Bethyl	A302-201A-T	WB	rabbit	p
25	N-Cadherin	Abcam	ab98952	WB	mouse	m
26	Nectin 2	Santa Cruz	sc-271236	WB	mouse	m
27	Occludin	Santa Cruz	sc-133256 (E-5)	WB	mouse	m
28	P53	Santa Cruz	sc-126	WB	mouse	m
29	pan-Rac	Santa Cruz	sc-514583	WB	mouse	m
30	PARP	Santa Cruz	sc-8007	WB	mouse	m
31	Paxillin	BD Biosciences	610051	WB	rabbit	m
32	Paxillin (phospho-Y118)	Cell Signaling	2541S	WB, IF	rabbit	m
33	P-Cadherin	Santa Cruz	sc-74545	WB	mouse	m
34	PRK1/2/3 (phospho-T774/816/718)	Cell Signaling	2611S	WB	rabbit	m
35	PSA	DAKO	A0562	WB	rabbit	p
36	RhoA	Santa Cruz	sc-418	WB	mouse	m
37	RhoB	Santa Cruz	sc-8048	WB	mouse	m
38	RhoC	Cell Signaling	3430S	WB, IF	rabbit	m
39	SRF	Cell Signaling	5147T	WB	rabbit	m
40	Thrombospondin 1	Santa Cruz	sc-393504	WB	mouse	m
41	Ube2F	Santa Cruz	sc-398668	WB	mouse	m
42	Vinculin	SIGMA	V9131	WB	mouse	m
43	YAP	Santa Cruz	sc-376830	WB	mouse	m
44	YBX3/CSDA	SIGMA	SAB1404593	IF	mouse	m
45	YBX3/ZONAB	Bethyl	A303-070A-T	WB	rabbit	p
46	ZO1	Invitrogen	10342463	WB, IF, IP	rabbit	p

Supplementary Table S3. List of siGENOME® SMARTpool and individual (shown in bold) siRNAs.

#	Pool Reference	Duplex Reference	Gene Symbol	GENE ID	Gene Accession	GI Number	Sequence
1	M-016305-01	D-016305-01	CACUL1	143384	NM_153810	109452596	UAAUAUAGCUCUUGGACAA
2	M-016305-01	D-016305-02	CACUL1	143384	NM_153810	109452596	CCAGAUCUCUAUAUUGAAA
3	M-016305-01	D-016305-03	CACUL1	143384	NM_153810	109452596	GAUGUAUAGUGAUCUGAUU
4	M-016305-01	D-016305-04	CACUL1	143384	NM_153810	109452596	UGGCAAAUAUUGUGAAAGG
5	M-015562-00	D-015562-01	CAND1	55832	NM_018448	21361793	GAAAGGAGCUUAUAAGAGA
6	M-015562-00	D-015562-02	CAND1	55832	NM_018448	21361793	UAACUGAGCUGGUAAAUGU
7	M-015562-00	D-015562-03	CAND1	55832	NM_018448	21361793	GCACUAACAUGCUUUCUGA
8	M-015562-00	D-015562-04	CAND1	55832	NM_018448	21361793	GAAAUUACCAGGUUAACUA
9	M-023448-01	D-023448-13	CAND2	23066	NM_012298	112420976	CUUAAAGAUCGGAGCGUCA
10	M-023448-01	D-023448-14	CAND2	23066	NM_012298	112420976	GGAAAUGGGUCCUCACGUG
11	M-023448-01	D-023448-15	CAND2	23066	NM_012298	112420976	GAAGACGAGUACAGCGAUG
12	M-023448-01	D-023448-16	CAND2	23066	NM_012298	112420976	CCGUCAUCACAGCGGUCAA
13	M-004086-01	D-004086-01	CUL1	8454	NM_003592	32307160	CGACAGCACUCAAAUUAAA
14	M-004086-01	D-004086-03	CUL1	8454	NM_003592	32307160	GGUUAUAUCAGUUGUCUAA
15	M-004086-01	D-004086-04	CUL1	8454	NM_003592	32307160	AGACUUGGAUUUCAGCAUU
16	M-004086-01	D-004086-05	CUL1	8454	NM_003592	32307160	CAACGAAGAGUUCAGGUUU
17	M-007277-00	**D-007277-01**	CUL2	8453	NM_003591	19482173	**GAAGGAAACUUACAUGGUU**
18	M-007277-00	**D-007277-02**	CUL2	8453	NM_003591	19482173	**GCGCUGAUCUCAACAAUAA**
19	M-007277-00	**D-007277-03**	CUL2	8453	NM_003591	19482173	**GGAAGUGCAUGGUAAAUUU**
20	M-007277-00	**D-007277-04**	CUL2	8453	NM_003591	19482173	**GGACAGCACUCAGAUGAAU**
21	M-010224-02	D-010224-03	CUL3	8452	NM_003590	45827792	CCGAACAUCUCAUAAAUAA
22	M-010224-02	D-010224-04	CUL3	8452	NM_003590	45827792	GAGAAGAUGUACUAAAUUC
23	M-010224-02	D-010224-05	CUL3	8452	NM_003590	45827792	GAGAUCAAGUUGUACGUUA
24	M-010224-02	D-010224-18	CUL3	8452	NM_003590	45827792	GCGGAAAGGAGAAGUCGUA
25	M-012610-01	D-012610-01	CUL4A	8451	NM_003589	57165422	GGAAGAGACUAAUUGCUUA
26	M-012610-01	D-012610-02	CUL4A	8451	NM_003589	57165422	GAACAGCGAUCGUAAUCAA
27	M-012610-01	D-012610-03	CUL4A	8451	NM_003589	57165422	GCAUGUGGAUUCAAAGUUA
28	M-012610-01	D-012610-04	CUL4A	8451	NM_003589	57165422	GAACCCAUAUUAUUAGUGA
29	M-017965-01	D-017965-01	CUL4B	8450	NM_001079872	121114301	GCUAUUGGCCGACAUAUGU
30	M-017965-01	D-017965-02	CUL4B	8450	NM_001079872	121114301	CAGAAGUCAUUAAUUGCUA
31	M-017965-01	D-017965-03	CUL4B	8450	NM_001079872	121114301	CAAACGGCCUAGCCAAAUC
32	M-017965-01	D-017965-04	CUL4B	8450	NM_001079872	121114301	CGGAAAGAGUGCAUCUGUA
33	M-019553-01	**D-019553-01**	CUL5	8065	NM_003478	67514034	**GACACGACGUCUUAUAUUA**
34	M-019553-01	**D-019553-02**	CUL5	8065	NM_003478	67514034	**CGUCUAAUCUGUUAAAGAA**
35	M-019553-01	**D-019553-03**	CUL5	8065	NM_003478	67514034	**GAUGAUACGGCUUUGCUAA**
36	M-019553-01	**D-019553-04**	CUL5	8065	NM_003478	67514034	**GUUCAACUACGAAUACUAA**
37	M-017673-00	D-017673-01	CUL7	9820	NM_014780	31543014	AGAAGCAGGUGAACAAUUU
38	M-017673-00	D-017673-02	CUL7	9820	NM_014780	31543014	GAACUCAACUCGGUGAAUG
39	M-017673-00	D-017673-03	CUL7	9820	NM_014780	31543014	GAACCUGCCUGCCCUCCUA
40	M-017673-00	D-017673-04	CUL7	9820	NM_014780	31543014	GAACUCCGCUACAGGGAAU
41	M-019139-01	D-019139-01	DCUN1D1	54165	NM_020640	36030882	CAGAUGACAUGUCUAAUUA
42	M-019139-01	D-019139-02	DCUN1D1	54165	NM_020640	36030882	GCAUUAGUGUGUUGAUUAU
43	M-019139-01	D-019139-03	DCUN1D1	54165	NM_020640	36030882	GCAGUAAGUUGUCUUUCUC
44	M-019139-01	D-019139-04	DCUN1D1	54165	NM_020640	36030882	GAACCAGGACGAUUUAAGG
45	M-020261-01	D-020261-01	DCUN1D2	55208	NM_001014283	62122951	UGACAGAACUUGGGUGUGA
46	M-020261-01	D-020261-02	DCUN1D2	55208	NM_001014283	62122951	CGAGAGAACUGCUAUCUAC
47	M-020261-01	D-020261-03	DCUN1D2	55208	NM_001014283	62122951	CAGAAUGAGUGGAGACUAG
48	M-020261-01	D-020261-13	DCUN1D2	55208	NM_001014283	62122951	CACUCAGGCUGGCGAGAGA
49	M-018390-01	D-018390-01	DCUN1D3	123879	NM_173475	27735046	GCAAAGCAAUAAGUGCAGA
50	M-018390-01	D-018390-02	DCUN1D3	123879	NM_173475	27735046	AAGGAUCUCUACCGGUUUA
51	M-018390-01	D-018390-03	DCUN1D3	123879	NM_173475	27735046	CAAGCCAGGUGGAGAUAUC
52	M-018390-01	D-018390-04	DCUN1D3	123879	NM_173475	27735046	CAGAACAAUCCUCCGGUAU
53	M-014118-02	**D-014118-01**	DCUN1D4	23142	NM_015115	94536779	**GGAGUGGUAUAAAGACAAA**
54	M-014118-02	**D-014118-02**	DCUN1D4	23142	NM_015115	94536779	**CGACUAGAAUAAAGACUGA**
55	M-014118-02	**D-014118-03**	DCUN1D4	23142	NM_015115	94536779	**GCAAUGUCCUAGAGUUUAG**
56	M-014118-02	**D-014118-04**	DCUN1D4	23142	NM_015115	94536779	**CUGAAGGCAUGGAGAAAUU**
57	M-014842-01	D-014842-01	DCUN1D5	84259	NM_032299	34147410	GAACAAAGAUCAAUGGUAC
58	M-014842-01	D-014842-02	DCUN1D5	84259	NM_032299	34147410	GCAGAACAGUCCAUGCUGA
59	M-014842-01	D-014842-03	DCUN1D5	84259	NM_032299	34147410	CAGAGAAGCCUUGAUAUUG
60	M-014842-01	D-014842-04	DCUN1D5	84259	NM_032299	34147410	CGCUCACAGUUGAAUGAUA
61	M-006401-02	D-006401-01	NAE1	8883	NM_001018160	66363687	GAUGAUCGCUGCAUAAAUA
62	M-006401-02	D-006401-04	NAE1	8883	NM_001018160	66363687	GUAAUCAUGUUGCCAAAUU
63	M-006401-02	D-006401-19	NAE1	8883	NM_001018160	66363687	UGAAACAAAUGGACGAAUA
64	M-006401-02	D-006401-20	NAE1	8883	NM_001018160	66363687	GGGUUGUGCUUUAGUCUGU
65	M-020081-01	D-020081-01	NEDD8	4738	NM_006156	148922866	GAAAGGAGAUUGAGAUUGA
66	M-020081-01	D-020081-02	NEDD8	4738	NM_006156	148922866	AGAUUGAGAUUGACAUUGA
67	M-020081-01	D-020081-03	NEDD8	4738	NM_006156	148922866	CAGACAAGGUGGAGCGAAU
68	M-020081-01	D-020081-04	NEDD8	4738	NM_006156	148922866	GGAGAUUGAGAUUGACAUU
69	M-004087-01	D-004087-01	RBX1	9978	NM_014248	22091459	GAAGCGCUUUGAAGUGAAA
70	M-004087-01	D-004087-03	RBX1	9978	NM_014248	22091459	GCAUAGAAUGUCAAGCUAA
71	M-004087-01	D-004087-05	RBX1	9978	NM_014248	22091459	GCAAGAAGCGCUUUGAAGU
72	M-004087-01	D-004087-06	RBX1	9978	NM_014248	22091459	CAACAGAGAGUGGGAAUUC
73	M-006907-02	D-006907-02	RNF7	9616	NM_014245	156071506	UCUUAGAUGUCAAGCUGAA
74	M-006907-02	D-006907-04	RNF7	9616	NM_014245	156071506	CCGACGUGGAAGACGGAGA
75	M-006907-02	D-006907-05	RNF7	9616	NM_014245	156071506	CCAAAGAAUCGGCAAAUGA
76	M-006907-02	D-006907-18	RNF7	9616	NM_014245	156071506	UGGGACGUGGAGUGCGAUA
77	M-005249-00	D-005249-01	UBA3	9039	NM_003968	38045941	GAGAGAGCAUCACAAUAUA
78	M-005249-00	D-005249-02	UBA3	9039	NM_003968	38045941	GCCAGAAGAUGGAUAAAUG
79	M-005249-00	D-005249-03	UBA3	9039	NM_003968	38045941	CAUUGGAGCUGGCGGCUUA
80	M-005249-00	D-005249-04	UBA3	9039	NM_003968	38045941	GGUAACCUCUAUUGAAGAA
81	M-009081-01	**D-009081-01**	UBE2F	140739	NM_080678	18087856	**GAAGCUAAUUUACCUUGUA**
82	M-009081-01	**D-009081-02**	UBE2F	140739	NM_080678	18087856	**GAGAGAACAUUCAAUUGAU**
83	M-009081-01	**D-009081-03**	UBE2F	140739	NM_080678	18087856	**UGAAAUGCCUGACCAAGAU**
84	M-009081-01	**D-009081-04**	UBE2F	140739	NM_080678	18087856	**UAAAGAGGUUGCAGAACUU**
85	M-004348-01	D-004348-01	UBE2M	9040	NM_003969	150417997	GAUGAGGGCUUCUACAAGA
86	M-004348-01	D-004348-02	UBE2M	9040	NM_003969	150417997	GAAGCCAGUCCUUACGAUA
87	M-004348-01	D-004348-03	UBE2M	9040	NM_003969	150417997	CAGACGACCUCCUCAACUU
88	M-004348-01	D-004348-04	UBE2M	9040	NM_003969	150417997	GAGCUGAACCUGCCCAAGA
89	M-003860-03	**D-003860-01**	RHOA	387	NM_001664	1519243696	**AUGGAAAGCAGGUAGAGUU**
90	M-003860-03	D-003860-02	RHOA	387	NM_001664	1519243696	GAACUAUGUGGCAGAUAUC
91	M-003860-03	D-003860-03	RHOA	387	NM_001664	1519243696	GAAAGACAUGCUUGCUCAU
92	M-003860-03	D-003860-04	RHOA	387	NM_001664	1519243696	GAGAUAUGGCAAACAGGAU
93	M-008395-04	D-008395-07	RHOB	388	NM_004040	1653962595	GCAUCCAAGCCUACGACUA
94	M-008395-04	**D-008395-08**	RHOB	388	NM_004040	1653962595	**ACACCGACGUCAUUCUCAU**
95	M-008395-04	D-008395-09	RHOB	388	NM_004040	1653962595	CAUCCAAGCCUACGACUAC
96	M-008395-04	D-008395-24	RHOB	388	NM_004040	1653962595	CAACUGCUGCAAGGUGCUA
97	M-008555-01	**D-008555-01**	RHOC	389	NM_175744	1779541909	**GGAGAGAGCUGGCCAAGAU**
98	M-008555-01	D-008555-02	RHOC	389	NM_175744	1779541909	AUAAGAAGGACCUGAGGCA
99	M-008555-01	D-008555-03	RHOC	389	NM_175744	1779541909	GGAUCAGUGCCUUUGGCUA
100	M-008555-01	D-008555-04	RHOC	389	NM_175744	1779541909	GAGAGCUGGCCAAGAUGAA
101	M-009800-02	**D-009800-01**	SRF	6722	NM_003131	1519314917	**GGACUGUGCUGAAGAGUAC**
102	M-015434-01	**D-015434-01**	MKL1	57591	NM_001282660	1677498929	**GGUGAACUAUCCCAAAGUA**
103	M-015434-01	D-015434-03	MKL1	57591	NM_001282660	1677498929	GGACAGAGGACUAUCUCAA
104	M-015434-01	D-015434-04	MKL1	57591	NM_001282660	1677498929	AAACUGAGCUGAUUGAGCG
105	M-015434-01	D-015434-17	MKL1	57591	NM_001282660	1677498929	CCAUGGACACCUCGGAAUU
106	M-012200-00	**D-012200-01**	YAP1	10413	NM_001282101	1890333929	**GGUCAGAGAUACUUCUUAA**
107	M-012200-00	D-012200-02	YAP1	10413	NM_001282101	1890333929	CCACCAAGCUAGAUAAAGA
108	M-012200-00	D-012200-03	YAP1	10413	NM_001282101	1890333929	GAACAUAGAAGGAGAGGAG
109	M-012200-00	D-012200-04	YAP1	10413	NM_001282101	1890333929	GCACCUAUCACUCUCGAGA
110	M-015793-00	**D-015793-01**	YBX3	8531	NM_003651	1519311901	**GGAAAUAUCUGCGCAGUGU**
111	M-015793-00	D-015793-02	YBX3	8531	NM_003651	1519311901	GGACAGACCUUUGACCGUC
112	M-015793-00	D-015793-03	YBX3	8531	NM_003651	1519311901	AGACGUGGCUACUAUGGAA
113	M-015793-00	D-015793-04	YBX3	8531	NM_003651	1519311901	CAACGUCAGAAAUGGAUAU
114	D-001206-13	**D-001210-01**	siGENOME Non-targeting Control	0		0	**UAGCGACUAAACACAUCAA**
115	D-001206-13	D-001210-02	siGENOME Non-targeting Control	0		0	UAAGGCUAUGAAGAGAUAC
116	D-001206-13	D-001210-03	siGENOME Non-targeting Control	0		0	AUGUAUUGGCCUGUAUUAG
117	D-001206-13	D-001210-04	siGENOME Non-targeting Control	0		0	AUGAACGUGAAUUGCUCAA

Supplementary Table S4. Regulation of Rho stability and activity by ubiquitylation- available data.

Rho	Rho-GTP<br>RBD-PD	Conditions	Ligase	Rho stability<br>(MLN, Pr-i, Lyso-i)	Ubiquitylation-evidence<br>Endogeneous-OE	 Native-Denaturing	 MLN ?	Degradation-proposed<br>Proteasome-Lysosome	Ref
A			Smurf-1	LLnL(+)	OE	Denaturing: IP 2x α-Flag-RhoA<br>WB α-HA-Ub		Proteasome	[18]
A		WT + NGF	Smurf-1	LLnL(+), lactacystin (+)<br>MG132 (+)	Endogeneous	Native: IP α-RhoA<br>WB α-Ub		Proteasome	[19]
A	yes	WT + CNF1<br>Q63L-mutant	Smurf-1		OE	Denaturing: PD his6-Ub<br>WB α-HA-RhoA		Proteasome	[20]
A			CRL3-BACURD	MG132 (+)	Endogeneous	Native: IP α-RhoA<br>WB α-Ub		Proteasome	[21]
A			CRL1-FBXL19	MG132 (+)	Endogeneous	Native: IP α-Ub<br>WB α-RhoA		Proteasome	[22]
A			CRL1-FBXL19	MG132 (+)	OE	Native: IP α-HA-RhoA<br>WB α-Ub		Proteasome	[23]
A	yes	TCIRG1-null<br>Q63L-mutant	?<br>autophagy	MG132 (NS), CQ (+)	Both	Native: IP α-RhoA<br>WB α-Ub & α-RhoA		Lysosome	[24]
A	yes		?	MLN (+), Btz (NS), MG132 (NS)<br>CQ (NS), NH4Cl (NS)				?	[25]
B	yes	WT<br>T19N-mutant	CRL3-KCTD10	MLN (+), Btz (+), MG132 (+)<br>CQ (+), NH4Cl (+)	Both	Native: IP α-RhoB<br>WB α-Ub<br>Denaturing: IP HA-Ub<br>WB α-RhoB	MLN (-)<br>Ub-conj.	Both	[25]
B			CRL3-KCTD10	MLN (+), MG132 (NS)<br>BafA1 (+)				Lysosome	[26]
B			Smurf-1	MG132 (+)	Both	Denaturing: IP 2x α-Flag-RhoA<br>WB α-HA-Ub<br>Native: IP α-HA-Ub<br>WB α-RhoB		Proteasome	[27]
B			CRL2	MLN (+), Btz (+)	Endogeneous	Native: IP α-RhoB<br>WB α-Ub	MLN (-)<br>Ub-conj.	Proteasome	[28]
C			?	MLN (+)				?	[25]
C		p38γ KD	?	MG132(NS), lactacystin (NS)<br>CQ (+), NH4Cl (+)	Endogeneous	Native: IP α-Ub<br>WB α-RhoC		Lysosome	[29]

Abbreviations: "Pr-i"- proteasome inhibitors, "Lyso-i" - lysosome inhibitors, "CQ"- chloroquine, "Btz"- bortezomib, "BafA1"- bafilomycin A1, "Endogenous"- the analysis of Rho ubiquitylation was performed at endogenous level, "OE"- a tagged version of Rho protein was over-expressed and used for ubiquitylation analysis, "IP"-immunoprecipitation, "PD"- pulldown, "WB"- western blotting, "Native"- Rho protein was isolated in non-denaturing conditions and could potentially be contaminated with other ubiquitylated proteins bound non-covalently to Rho, "Denaturing"- Rho protein was isolated in denaturing conditions that allowed non-ambiguous identification of Rho ubiquitylation, "(+)"- the drug increased the level of Rho protein, "(NS)" - the drug effect was non-significant, "MLN(-)"- MLN decreased the level of protein ubiquitylation as expected.

References: [18], [19], [20], [21], [22], [23], [24], [25], [26], [27], [28], [29].

Supplementary References

1. Rulina, A. V. *et al.* Distinct outcomes of CRL–Nedd8 pathway inhibition reveal cancer cell plasticity. *Cell Death & Disease* **7**, e2505–e2505 (2016).
2. Bhajun, R. *et al.* A statistically inferred microRNA network identifies breast cancer target miR-940 as an actin cytoskeleton regulator. *Scientific Reports* **5**, 8336 (2015).
3. Haguet, V. *et al.* Time-lapse screening by parallelized lensfree imaging. *Proc. 17th International Conference on Miniaturized Systems for Chemistry and Life Sciences (MicroTAS)* 1740–1742 (2013).
4. Ghenim, L. *et al.* A new ultradian rhythm in mammalian cell dry mass observed by holography. *Scientific Reports* **11**, (2021).
5. Tinevez, J.-Y. *et al.* TrackMate: An open and extensible platform for single-particle tracking. *Methods* **115**, 80–90 (2017).
6. Mittler, F. *et al.* High-Content Monitoring of Drug Effects in a 3D Spheroid Model. *Frontiers in Oncology* **7**, (2017).
7. Takahashi, A. *et al.* Role of C-terminal regions of the C-terminal fragment of Clostridium perfringens enterotoxin in its interaction with claudin-4. *Journal of Controlled Release* **108**, 56–62 (2005).
8. Sebbagh, M. *et al.* Caspase-3-mediated cleavage of ROCK I induces MLC phosphorylation and apoptotic membrane blebbing. *Nature cell biology* **3**, 346–352 (2001).
9. Augspach, A. *et al.* Activation of RhoA,B,C by Yersinia Cytotoxic Necrotizing Factor (CNFy) Induces Apoptosis in LNCaP Prostate Cancer Cells. *Toxins* **5**, 2241–2257 (2013).
10. Ren, X.-D. Regulation of the small GTP-binding protein Rho by cell adhesion and the cytoskeleton. *The EMBO Journal* **18**, 578–585 (1999).
11. Bompard, G., Sharp, S. J., Freiss, G. & Machesky, L. M. Involvement of Rac in actin cytoskeleton rearrangements induced by MIM-B. *Journal of cell science* **118**, 5393–5403 (2005).
12. Stofega, M., DerMardirossian, C. & Bokoch, G. M. Affinity-Based Assay of Rho Guanosine Triphosphatase Activation. in *Transmembrane Signaling Protocols* vol. 332 269–280 (Humana Press, 2006).
13. Ren, X.-D. & Schwartz, M. A. Determination of GTP loading on Rho. in *Methods in Enzymology* vol. 325 264–272 (Elsevier, 2000).
14. Balda, M. S. & Matter, K. The tight junction protein ZO-1 and an interacting transcription factor regulate ErbB-2 expression. *The EMBO Journal* **19**, 2024–2033 (2000).
15. Wilson, M. D., Saponaro, M., Leidl, M. A. & Svejstrup, J. Q. MultiDsk: A Ubiquitin-Specific Affinity Resin. *PLoS ONE* **7**, e46398 (2012).
16. Holden, P. & Horton, W. A. Crude subcellular fractionation of cultured mammalian cell lines. *BMC Research Notes* **2**, 243 (2009).
17. Watari, A., Hashegawa, M., Muangman, T., Yagi, K. & Kondoh, M. Use of cell-based screening to identify small-molecule compounds that modulate claudin-4 expression. *Biotechnology Letters* **37**, 1177–1185 (2015).
18. Wang, H.-R. Regulation of Cell Polarity and Protrusion Formation by Targeting RhoA for Degradation. *Science* **302**, 1775–1779 (2003).
19. Deglincerti, A. *et al.* Coupled local translation and degradation regulate growth cone collapse. *Nature Communications* **6**, (2015).
20. Boyer, L. *et al.* CNF1-induced Ubiquitylation and Proteasome Destruction of Activated RhoA Is Impaired in Smurf1-/- Cells. *Molecular Biology of the Cell* **17**, 9 (2006).
21. Chen, Y. *et al.* Cullin Mediates Degradation of RhoA through Evolutionarily Conserved BTB Adaptors to Control Actin Cytoskeleton Structure and Cell Movement. *Molecular Cell* **35**, 841–855 (2009).
22. Wei, J. *et al.* A new mechanism of RhoA ubiquitination and degradation: Roles of SCF FBXL19 E3 ligase and Erk2. *Biochimica et Biophysica Acta (BBA) - Molecular Cell Research* **1833**, 2757–2764 (2013).
23. Li, H. *et al.* Fbxw7 regulates tumor apoptosis, growth arrest and the epithelial-to-mesenchymal transition in part through the RhoA signaling pathway in gastric cancer. *Cancer Letters* **370**, 39–55 (2016).
24. Belaid, A. *et al.* Autophagy Plays a Critical Role in the Degradation of Active RHOA, the Control of Cell Cytokinesis, and Genomic Stability. *Cancer Research* **73**, 4311–4322 (2013).
25. Kovačević, I. *et al.* The Cullin-3–Rbx1–KCTD10 complex controls endothelial barrier function via K63 ubiquitination of RhoB. *Journal of Cell Biology* **217**, 1015–1032 (2018).
26. Murakami, A. *et al.* Cullin-3/KCTD10 E3 complex is essential for Rac1 activation through RhoB degradation in human epidermal growth factor receptor 2-positive breast cancer cells. *Cancer Science* **110**, 650–661 (2019).
27. Wang, M. *et al.* ATR/Chk1/Smurf1 pathway determines cell fate after DNA damage by controlling RhoB abundance. *Nat Commun* **5**, 4901 (2014).
28. Xu, J. *et al.* The Neddylation-Cullin 2-RBX1 E3 Ligase Axis Targets Tumor Suppressor RhoB for Degradation in Liver Cancer. *Molecular & Cellular Proteomics* **14**, 499–509 (2015).
29. Rosenthal, D. T. *et al.* p38γ Promotes Breast Cancer Cell Motility and Metastasis through Regulation of RhoC GTPase, Cytoskeletal Architecture, and a Novel Leading Edge Behavior. *Cancer Research* **71**, 6338–6349 (2011).
